# Hepatitis C Virus Epidemiology in Djibouti, Somalia, Sudan, and Yemen: Systematic Review and Meta-Analysis

**DOI:** 10.1371/journal.pone.0149966

**Published:** 2016-02-22

**Authors:** Karima Chaabna, Silva P. Kouyoumjian, Laith J. Abu-Raddad

**Affiliations:** 1 Infectious Disease Epidemiology Group, Weill Cornell Medical College in Qatar, Cornell University, Qatar Foundation – Education City, Doha, Qatar; 2 Department of Healthcare Policy and Research, Weill Cornell Medical College, Cornell University, New York, New York, United States of America; 3 College of Public Health, Hamad bin Khalifa University, Doha, Qatar; University of Cincinnati College of Medicine, UNITED STATES

## Abstract

**Objectives:**

To characterize hepatitis C virus (HCV) epidemiology and assess country-specific population-level HCV prevalence in four countries in the Middle East and North Africa (MENA) region: Djibouti, Somalia, Sudan, and Yemen.

**Methods:**

Reports of HCV prevalence were systematically reviewed as per PRISMA guidelines. Pooled HCV prevalence estimates in different risk populations were conducted when the number of measures per risk category was at least five.

**Results:**

We identified 101 prevalence estimates. Pooled HCV antibody prevalence in the general population in Somalia, Sudan and Yemen was 0.9% (95% confidence interval [95%CI]: 0.3%–1.9%), 1.0% (95%CI: 0.3%–1.9%) and 1.9% (95%CI: 1.4%–2.6%), respectively. The only general population study from Djibouti reported a prevalence of 0.3% (CI: 0.2%–0.4%) in blood donors. In high-risk populations (e.g., haemodialysis and haemophilia patients), pooled HCV prevalence was 17.3% (95%CI: 8.6%–28.2%) in Sudan. In Yemen, three studies of haemodialysis patients reported HCV prevalence between 40.0%-62.7%. In intermediate-risk populations (e.g.. healthcare workers, in patients and men who have sex with men), pooled HCV prevalence was 1.7% (95%CI: 0.0%–4.9%) in Somalia and 0.6% (95%CI: 0.4%–0.8%) in Sudan.

**Conclusion:**

National HCV prevalence in Yemen appears to be higher than in Djibouti, Somalia, and Sudan as well as most other MENA countries; but otherwise prevalence levels in this subregion are comparable to global levels. The high HCV prevalence in patients who have undergone clinical care appears to reflect ongoing transmission in clinical settings. HCV prevalence in people who inject drugs remains unknown.

## Introduction

The global distribution of hepatitis C virus (HCV) infection is the consequence of national and local circumstances that have facilitated or limited HCV transmission in different populations [1–3]. The geographical distribution of this infection appears to vary from one region to another. The Middle East and North Africa (MENA) region appears to have the highest HCV prevalence worldwide [4, 5] with Egypt recording the highest national prevalence in the adult population at 14.7% [6, 7]. While the epidemiology of this infection is well studied in Egypt [6, 7], the infection status in most other MENA countries is yet to be well understood.

By applying a methodology developed recently [8, 9], this study aims to characterize the epidemiology of HCV infection and to estimate the national population-level HCV antibody prevalence in Djibouti, Somalia, Sudan, and Yemen, a group of MENA countries that we have labelled conventionally as the Horn of Africa subregion of MENA. This group of MENA countries were studied within the framework of one study because of their geographic proximity. This study is part of a larger ongoing project—the MENA HCV Epidemiology Synthesis Project [7–14]–that aims to characterize the epidemiology of HCV across the MENA region and to inform public health policy and programming at the national and regional levels.

## Materials and Methods

The protocol for this systematic review has been described elsewhere [8] and is registered at the International Prospective Register of Systematic Reviews under registration number CRD42014010318 [9]. The study methodology of the present article was also applied and refined in several previous studies of HCV epidemiology in different subregions and countries within MENA [7, 12–14]. We summarize our methodology in the following subsections. Further details can be found in the earlier descriptions and applications of this methodology [7–9, 12–14].

### Data sources and search strategy

This review was conducted based on the items outlined in the Preferred Reporting Items for Systematic Reviews and Meta-Analyses (PRISMA) statement [15] (S1 Table). The search criteria are provided in S2 Table. As in previous studies [7, 12–14], we searched for English and non-English reports in PubMed, Embase and the World Health Organization (WHO) regional databases (WHO African Index Medicus [16] and WHO Index Medicus for the Eastern Mediterranean Region [17]) for entries up to May 17^th^, 2015. To identify further relevant reports, we screened all articles archived in online national scientific journals not indexed in PubMed or Embase (up to May 17^th^, 2015). These journals included the Yemeni Journal of Medical Sciences [18], the Sudan Journal of Medical Sciences [19] and the Sudan Medical Journal [20]. Moreover, the literature database of the MENA HIV/AIDS Epidemiology Synthesis Project was searched for potentially relevant country-level and international organizations’ reports (up to April 14, 2015) [21, 22].

The database search was supplemented by checking references of the included reports and identified reviews. Lastly, we also searched the conference archives of the International AIDS Society conferences [23] and the ‘Endemic and Emerging Viral Diseases of Priority in the Middle East and North Africa (MENA)” workshop [24] (up to May 17^th^, 2015).

### Inclusion and exclusion criteria

The inclusion and exclusion criteria were developed along the lines of our previous systematic reviews [7, 12–14]. Studies with primary data were eligible for inclusion if they included populations who are permanently residing in any of the countries of Djibouti, Somalia, Sudan and Yemen. Our systematic review did not include migrants from these countries who are residing elsewhere. Studies such as Shire et *al*. 2012 [25] among migrant Somalis in the United States did not fit our inclusion criteria. Our definition for the MENA region was based on the MENA definition of the Eastern Mediterranean Regional Office of the World Health Organization (EMRO/WHO), the Joint United Nations Programme on HIV/AIDS (UNAIDS), and the World Bank. Djibouti, Somalia, Sudan, and Yemen were grouped together as part of one study because of their geographic proximity and because they could not fit within the scope of the other sub-regional studies of the MENA HCV Epidemiology Synthesis Project [7, 10–14]. Although these countries were grouped in one article, we conducted the epidemiological analyses for each country separately. Sudan and South Sudan were considered as one country because the time period covered in this review extends well beyond the independence of South Sudan in 2011.

Other criteria for inclusion were availability of data on serological testing for HCV antibody and an estimate of HCV incidence or prevalence. Reports were considered ineligible if they were based on self-report, saliva testing, or classification of HCV as non-A non-B hepatitis. Case reports, case series, reviews, qualitative studies, editorials, commentaries, letters to editors, author replies and animal studies were excluded. Reports were included in the systematic review if the study sample size was greater than 15. In the meta-analyses however, we included from the systematic review studies only the studies that had a sample size greater than 25 for consistency with the other sub-regional analyses [10–14].

### Study selection

**Fig 1:**
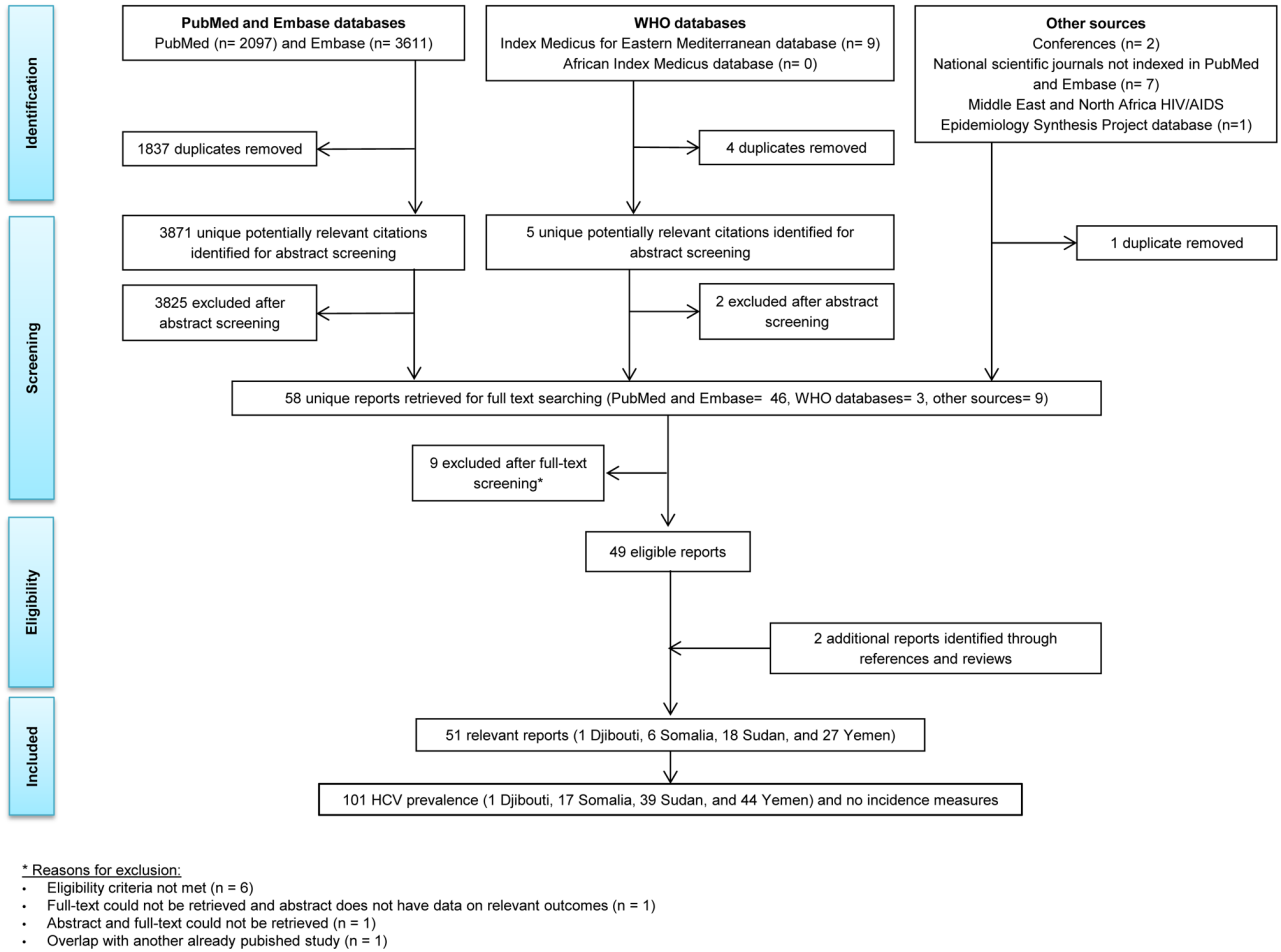
Flow of report selection for HCV incidence and prevalence in Djibouti, Somalia, Sudan and Yemen, adapted from the PRISMA guidelines [15].

References obtained through the search strategy were imported into a reference manager, Endnote [26], where duplicate reports were identified and excluded. The titles and abstracts of identified unique reports were screened for relevance by Karima Chaabna (KC). After title and abstract screening, full texts of the reports deemed relevant or potentially relevant were retrieved and assessed for eligibility for inclusion by KC. During this step, any remaining non-eligible reports were excluded and the reasons for their exclusion recorded (Fig 1).

#### Data extraction and synthesis

Relevant data were extracted by KC and checked for correctness by Silva Kouyoumjian (SK). Discrepancies were discussed and resolved by the study team. As in previous studies [7, 12–14], extracted data included reference details (author(s), year of publication, title, and journal), study location, year(s) of data collection, study design, sampling technique, population profile (such as blood donors and healthcare workers (HCWs)), socio-demographic characteristics of the population (sex and age), number of participants who were included and who did participate, response rate, name of the serological test used to determine HCV exposure, and raw results obtained for the primary outcome (HCV incidence or prevalence estimates). The following parameters were also recorded from included studies when available: HCV RNA incidence and prevalence estimates; HCV genotype frequency; and adjusted or unadjusted statistically significant (probability (*p)* value ≤0.05) risk factors for HCV exposure. Relevant data were extracted from abstracts for studies for which full texts could not be obtained even after contacting the authors. Decimal places of prevalence figures were reported in this article as reported in the original reports. Prevalence figures with more than one decimal place were rounded to one decimal place, with the exception of those below 0.1%. All meta-analyses used original raw numbers of cases and samples, with no rounding.

In this systematic review, the word ‘report’ refers to a publication (article, country-level report, or conference abstract, among others) that presents one or several outcome measures, while the word ‘study’ refers to any one specific outcome measure. Several reports of the same outcome measure were identified as duplicates and counted as one study. HCV measures were treated and counted as separate studies if, within the same report, HCV measures were reported stratified by population subgroup, and/or sex, and/or study period, and/or region, and/or age.

As in the earlier studies [7, 12–14], extracted data were presented and analysed stratified by country and according to the study population’s risk of acquiring HCV infection as follows:

1. Populations at high risk: these include patients with a history of haemodialysis (HD), thalassaemia, haemophilia, multiple transfusions, or schistosomiasis. In addition, these high-risk populations included people who inject drugs (PWID).
2. Populations at intermediate risk: these include HCWs, patients with sexually transmitted diseases (STDs), hospitalized patients, female sex workers (FSWs), and men who have sex with men (MSM).
3. Populations at low-risk (general populations): these include pregnant women, antenatal clinic attendees, blood donors, tuberculosis outpatients, military personnel, controls in case-control studies, and healthy adults and children among other general populations.
4. Special clinical populations: these include patients with specific diseases that could be related to HCV infection such as chronic liver disease (CLD), viral hepatitis, cirrhosis, hepatocellular carcinoma (HCC), and non-Hodgkin lymphoma (NHL). This category also includes other clinical populations who could have been exposed to HCV in clinical settings, such as patients with leprosy or those treated in surgical departments, but at variable risk of exposure. In essence, this category encompasses patients with clinical conditions associated with HCV infection or patients at risk of exposure to HCV in clinical settings but with an uncertain level of exposure making it difficult to classify them under the former three risk population groups.
5. Mixed populations: these comprise samples of persons with variable HCV infection risk that is a mix of the other four population groups.

### Quantitative analysis

Data analyses were conducted in R v.3.1.1. [27] using the *meta* package [28] and in Stata/SE 13.1 using the *metan* command [29]. The 95% confidence interval (95%CI) for HCV prevalence estimate in each individual included study was calculated using the Clopper-Pearson (binomial) method [30]. Studies presenting a minimum sample size of 25 participants were included in the meta-analysis. HCV prevalence estimates were pooled when at least five studies were included in each risk population category for each country. All meta-analyses were conducted using random-effects models, to account for expected heterogeneity in effect size across studies. HCV prevalence estimates were weighed by the inverse-variance of the double-arcsine transformed proportions [31], according to the method described by DerSimonian and Laird [32]. The back-transformed pooled mean proportions were calculated using Miller’s inverse transformation with the harmonic mean of the sample sizes [33]. The value 0.5 was added to all cell frequencies of studies with a zero cell count [34]. Sensitivity analysis was conducted using the value of 0.01, instead of 0.5, but the same mean proportions and their 95%CIs were obtained. Forest plots for all meta-analyses were generated.

To assess heterogeneity across studies, forest plots were inspected visually and Cochran’s Q test was conducted [35]. A two-sided Q test *p*-value of <0.10 was considered as significant. I^2^ heterogeneity measure and its 95%CI were calculated to assess the magnitude of between-study variation that is due to heterogeneity in effect size rather than chance [36]. The prediction interval was calculated to describe the distribution of true effects around the mean [37–39]. In situations of high heterogeneity and potential non-random biases, such as possibly in HCV prevalence measures in a given risk population, the prediction interval may provide a more interpretable summary of the variation in effect size in existing studies and potential true population mean.

A meta-regression [40] was undertaken to identify study-level factors contributing to the between-study heterogeneity in the pooled mean general population prevalence estimate. The following factors were entered into univariable and multivariable models: country, subpopulation within the low risk population, study design, study site, and precision of the measure. These factors were selected based on being reported in at least ten studies. These factors were included in the multivariable model if the p-value was <0.10.

National population-level HCV prevalence was estimated using individual study estimates of HCV prevalence in the general population in each country. Sensitivity analyses were conducted to assess the impact of excluding blood donor studies on pooled mean HCV prevalence estimates, and to assess the impact of excluding studies with a sample size lower than 1,000 and those published before 2000. The latter sensitivity analysis was motivated by the inclusion criteria in global estimations for HCV prevalence conducted recently by Gower *et al*.[41].

### Quality assessment

Per earlier developed methodology [8, 9], the quality of individual HCV prevalence estimates was determined by assessing the risk of bias (ROB) for each study and by evaluating the precision of each reported measure. ROB assessment was conducted by assessing the sources of bias that may affect the pooled mean estimates. Based on the Cochrane approach [40], each HCV prevalence measure was classified as having a low, high, or unclear ROB in three domains: sampling methodology, HCV infection ascertainment, and response rate. Response rate was defined as the number of tested individuals divided by the number of all persons invited to participate in the study [42]. ROB was considered low if (1) sampling was probability-based, (2) HCV was ascertained by biological assays, and (3) response rate was ≥80% or, for studies using respondent-driven sampling, ≥80% of the target sample size was reached. Studies with missing information for a specific domain were classified as having unclear ROB for that specific domain.

A study HCV prevalence estimate was considered as having good precision if the number of HCV tested individuals was at least 100. For a median HCV prevalence in the general population in this MENA subregion of 1.3% (see Results), this implies a 95%CI of 0%-5%.

## Results

### Search results

The process of study selection was based on PRISMA guidelines [15] (Fig 1). We identified a total of 5,708 citations: 2,097 through PubMed, 3,611 through Embase, nine through WHO databases, two through conference databases, seven through national scientific journals not indexed in PubMed or Embase, and one through the MENA HIV/AIDS Epidemiology Synthesis Project database. A total of 58 records were identified as relevant or potentially relevant after removing duplicates and screening the titles and abstracts of remaining records. Out of these 58 records, 49 were eligible for inclusion in the systematic review at the full-text screening stage. The remaining were excluded for multiple reasons such as not meeting the eligibility criteria, absence of data on relevant indicators in the full text and/or abstract, failure to retrieve the full text and/or abstract of a record, and duplication of another study included in the review. Two additional records were identified through screening the bibliographies of studies and reviews [43, 44]. To sum up, a total of 51 eligible reports and 101 HCV prevalence studies were included in the systematic review. These included one study in Djibouti, 17 studies in Somalia, 39 studies in Sudan and 44 studies in Yemen. No HCV incidence study was identified in this subregion.

### HCV prevalence overview

HCV prevalence ranged from 2.2% to 62.7% (number of studies (n) = 11 and median = 18.4%) in high-risk populations and from 6.3% to 40.3% (n = 17 and median = 17.8%) in special clinical populations. Meanwhile, HCV prevalence ranged from 0% to 5.8% (n = 31 and median = 0.5%) in intermediate-risk populations and from 0% to 8.5% (n = 42 and median = 1.3%) in general populations. In what follows we present an overview of country-level HCV prevalence studies.

#### Djibouti

The single study from Djibouti reported a prevalence of 0.3%. The majority of the sample were male blood donors (17–65 years old) [43] (Table 1).

**Table 1:** Studies reporting HCV prevalence in the general population in Djibouti, Somalia, Sudan and Yemen.

Author, Year	Location	Study period	Study design	Population characteristic	Sample size	Prevalence (%)*
**Djibouti**						
Dray, 2005 (43)	Djibouti	1998–2000	Cross-sectional	Blood donors	8,057	0.3
**Somalia**						
Bile, 1992 (48)	Mogadishu	1987	Cross-sectional	Children in government-operated residence for abandoned children in SOS institution (boy and girls)	76	0
EMRO, 2011 (49)	National	-	Cross-sectional	Blood donors	12,759	0.5
Nur, 2000 (45)	Mogadishu	1995	Cross-sectional	Blood donors	157	0.6
Aceti, 1993 (44)	Mogadishu	1988–1990	Retrospective	Blood donors (nursing school students) no history of parenteral exposure to blood or blood products, nor clinical or pathological picture compatible with viral hepatitis, nor any other apparent indication of liver disease.	309	1
Bile, 1992 (48)	Mogadishu	1987	Cross-sectional	Children in government-operated residence for abandoned children in Shebeli (girls)	287	1
Watts, 1994 (46)	Mogadishu, Merca and Chismayu	1990	Cross-sectional	Military personnel	79	1.3
Bile, 1992 (48)	Mogadishu	1987	Cross-sectional	Children in government-operated residence for abandoned children in Shebeli (boys)	309	1.9
Watts, 1994 (46)	Mogadishu, Merca and Chismayu	1990	Cross-sectional	Outpatients with tuberculosis	43	2.3
Bile, 1993 (47)	Mogadishu	1989	Case-control	Control patients treated from different hospital departments	62	6.4
**Sudan**						
Elfaki, 2008 (66)	El Obeid	-	Cross-sectional	Blood donors (farmers, shepherds, soldiers, lorry drivers, labours, employees, and others)	260	0
Elsheikh, 2007 (63)	Omdurman	2006	Cross-sectional	Pregnant women	423	0.6
Abou, 2009 (64)	Nyala (Dar Fur)	2007	Cross-sectional	Blood donors	400	1
Osman, 2014 (67)	Gezira	2011	Cross-sectional	Women attending maternity hospital (patients with antepartum haemorrhage, hypertension and diabetes mellitus excluded)	396	1.3
Nagi, 2007 (65)	Shendi	2005	Cross-sectional	Blood donors (males)	78	1.3
Omer, 2001 (61)	Gezira and North Kordofan	1996–1998	Case-control	Control from general population	199	2
Mohamedani, 2014 (50)	Gezira	-	Case-control	Non-*Schistosoma* infected controls	100	4
**Yemen**						
Gray, 1999 (89)	Hajjah	1992	Cross-sectional	General population	253	0
Sallam, 2003 (84)	Sana'a	1999–2002	Cross-sectional	Blood donors (males)	493	0.2
Sallam, 2003 (84)	Aden	1999–2002	Cross-sectional	Blood donors (males)	494	0.6
Saghir, 2012 (92)	Hobeidah	2009–2010	Cross-sectional	Blood donors	564	0.7
Selm, 2010 (69)	Aden	2007	Case-control	Control consisting of blood donors (males)	100	0.8
Saghir, 2012 (92)	Hobeidah	2008–2009	Cross-sectional	Blood donors	919	0.9
Gray, 1999 (89)	Ibb	1992	Cross-sectional	General population from Al Homaadi village	175	1
Gray, 1999 (89)	Ibb	1992	Cross-sectional	General population from Mouthan village	158	1
Oshaish, 2008 (91)	Taiz	-	Cross-sectional	Blood donors	1,000	1
El Guneid, 1993 (80)	Taiz	-	Cross-sectional	Blood donors (males)	294	1
Al-Waleedi, 2012 (83)	Aden	2007–2008	Cross-sectional	Blood donors	469	1.3
Gacche, 2012 (94)	Ibb	2010–2011	Cross-sectional	Healthy subjects	2,379	1.3
Sallam, 2003 (84)	Sana'a	1999–2002	Cross-sectional	African migrant community living in a shantytown	593	1.3
Omer, 2010 (88)	Aden	2007	Cross-sectional	Blood donors	5,825	2
Haidar, 2002 (68)	Hajjah	1997–1999	Cross-sectional	Blood donors	2,434	2
Al-Shamiri, 2011 (87)	Taiz	2007–2009	Cross-sectional	Children at school	141	2.1
Alodini, 2012 (90)	Sana'a	2010	Cross-sectional	Blood donors	3,000	3
El Guneid, 1993 (80)	Taiz	-	Cross-sectional	Healthy pregnant women	243	3.3
Salem, 2009 (78)	Sana'a	2005–2007	Case-control	Control patients—females—treated from different hospital departments (patients coming from different parts of the country)	8,055	3.5
Denis, 1994 (73)	.	1988–1990	Case-control	Control patients (blood donors and pregnant women)	51	3.9
Al-Moslih, 2001 (76)	Sana'a	-	Case-control	Control patients (no history of liver disease)	120	4.2
Salem, 2009 (78)	Sana'a	2005–2007	Case-control	Control patients—males—treated from different hospital departments (patients coming from different parts of the country)	20,329	4.3
Sallam, 2003 (84)	Soqotra Island	1999–2002	Cross-sectional	Residents of Soqotra	99	5
Scott, 1992 (86)	Sana'a, Hajjah, Taiz and Hobeidah	1988	Cross-sectional	Healthy children and adults	348	6
Murad, 2013 (85)	Sana'a	2011	Cross-sectional	Pregnant women at a hospital	400	8.5

*Prevalence figures with more than one decimal place were rounded to one decimal place.Abbreviations: SOS: Société Organisation Sociale; EMRO: WHO regional office for the Eastern Mediterranean.

#### Somalia

There were no studies in high risk populations. Among intermediate-risk populations, HCV prevalence was in the range of 0%–7% (n = 5 and median = 2.4%) [44–46] (Table 2). The highest HCV prevalence of 7% was recorded for hospitalized adults and the lowest of 0% was reported among hospitalized children [44]. In special clinical populations, HCV prevalence was high at 14.5% [44] and 40.3% [47] among patients with CLD (Table 2).

**Table 2:** Studies reporting HCV prevalence in high-risk, intermediate-risk and special clinical population groups in Djibouti, Somalia, Sudan and Yemen.

Population risk group	Author, Year	Location	Study period	Study design	Population characteristic	Sample size	Prevalence (%)*
**Somalia**							
Intermediate-risk	Aceti, 1993 (44)	Mogadishu	1988–1990	Cross-sectional	Hospitalized children at a hospital for diseases other than hepatitis	287	0
	Watts, 1994 (46)	Mogadishu, Merca and Chismayu	1990	Cross-sectional	Female sex workers	236	1.7
	Groen, 2000 (45)	Mogadishu	1995	Cross-sectional	Hospitalized children for measles, tuberculosis, anaemia and other febrile illnesses	42	2.4
	Watts, 1994 (46)	Mogadishu, Merca and Chismayu	1990	Cross-sectional	Patients with STDs	80	2.5
	Groen, 2000 (45)	Mogadishu	1995	Cross-sectional	Hospitalized adult for tuberculosis, malaria, acute respiratory infections, and unknown diagnosis (no clinically evident case of hepatitis)	57	7
Special Clinical populations	Aceti, 1993 (44)	Mogadishu	1988–1990	Cross-sectional	Patients with CLD	110	14.5
	Bile, 1993 (47)	Mogadishu	1989	Case-control	Cases with CLD including HCC	62	40.3
Mixed populations	Aceti, 1993 (44)	Mogadishu	1988–1990	Cross-sectional	Mixed population with high prevalence of *Schistosoma haematobium*: 98 prisoners and 81 patients from the Psychiatric Clinic of Mogadishu	179	2.2
**Sudan**							
High risk	Mudawi, 2007b (51)	Khartoum	2001	Cross-sectional	Patients with hepatosplenic schistosomiasis	176	4.5
	Gasim, 2012 (55)	Khartoum	2010	Cross-sectional	Haemodialysis patients	353	8.5
	Gadour, 2011 (53)	Khartoum	2008	Cross-sectional	Patients (males) with haemophilia	62	13
	El-Amin, 2007 (54)	Khartoum	2005	Cross-sectional	Haemodialysis patients	236	23.7
	Mohamedani, 2014 (50)	Gezira	-	Cross-sectional	Patients with schistosomiasis	106	31.1
	Suliman, 1995 (56)	Khartoum	1994	Cross-sectional	Haemodialysis patients	46	34.9
Mixed populations	Mudawi, 2007a (52)	Gezira	2000	Cross-sectional	Participant enrolled from the population of Um Zukra village (population schistosomiasis prevalence of 70%)	410	2.2
Intermediate-risk	IBBS National Team, 2013 (57)	Gezira	2011–2012	Cross-sectional	Female sex workers	296	0
	IBBS National Team, 2013 (57)	Khartoum	2011–2012	Cross-sectional	Men who have sex with men	292	0
	El-Amin, 2007 (54)	Khartoum	2005	Cross-sectional	Employees at a haemodialysis centre	62	0
	IBBS National Team, 2013 (57)	North Kodofan	2011–2012	Cross-sectional	Female sex workers	296	0
	Nail, 2008 (58)	Omdurman	2007	Cross-sectional	Health care workers at the Tropical Diseases Teaching Hospital	211	0
	IBBS National Team, 2013 (57)	River Nile	2011–2012	Cross-sectional	Female sex workers	291	0
	IBBS National Team, 2013 (57)	Sinnar	2011–2012	Cross-sectional	Men who have sex with men	312	0
	IBBS National Team, 2013 (57)	White Nile	2011–2012	Cross-sectional	Female sex workers	288	0
	IBBS National Team, 2013 (57)	North Kodofan	2011–2012	Cross-sectional	Men who have sex with men	304	0.2
	IBBS National Team, 2013 (57)	Sinnar	2011–2012	Cross-sectional	Female sex workers	303	0.2
	IBBS National Team, 2013 (57)	Alshamalia	2011–2012	Cross-sectional	Men who have sex with men	305	0.5
	IBBS National Team, 2013 (57)	Khartoum	2011–2012	Cross-sectional	Female sex workers	305	0.5
	IBBS National Team, 2013 (57)	West Darfur	2011–2012	Cross-sectional	Female sex workers	303	0.5
	IBBS National Team, 2013 (57)	River Nile	2011–2012	Cross-sectional	Men who have sex with men	300	0.6
	IBBS National Team, 2013 (57)	Gezira	2011–2012	Cross-sectional	Men who have sex with men	135	1
	IBBS National Team, 2013 (57)	White Nile	2011–2012	Cross-sectional	Men who have sex with men	307	1.1
	IBBS National Team, 2013 (57)	Alshamalia	2011–2012	Cross-sectional	Female sex workers	305	1.5
	Mudawi, 2014 (59)	Khartoum	2010–2012	Cross-sectional	Patients with HIV	358	1.7
	IBBS National Team, 2013 (57)	North Darfur	2011–2012	Cross-sectional	Female sex workers	303	2.6
	McCarthy, 1994 (60)	Juba	1989	Cross-sectional	Paediatric and adolescent patients	666	3
	IBBS National Team, 2013 (57)	South Darfur	2011–2012	Cross-sectional	Female sex workers	299	5.1
	Suliman, 1995 (56)	Khartoum	1994	Cross-sectional	Health care workers at the Khartoum Kidney Dialysis Center	37	5.4
	IBBS National Team, 2013 (57)	South Darfur	2011–2012	Cross-sectional	Men who have sex with men	172	5.9
Special Clinical populations	Ahmed, 2008 (62)	Khartoum	2007	Cross-sectional	Pregnant women with acute viral hepatitis at three main hospitals in Khartoum	16	6.3
	Omer, 2001 (61)	Khartoum	1996–1998	Case-control	Patients with HCC	115	11
**Yemen**							
High-risk	Haidar, 2002 (68)	Hajjah	1997–1999	Cross-sectional	Haemodialysis patients	30	40
	Aman, 2015 (70)	Aden	2000–2013	Cross-sectional	Haemodialysis patients	219	40.2
	Selm, 2010 (69)	Aden	2007	Case-control	Haemodialysis patients	51	62.7
Intermediate-risk	Haidar, 2002 (68)	Hajjah	1997–1999	Cross-sectional	Hospital employees	200	0.5
	Al-Jarba, 2003	Aden	-	Cross-sectional	Hospital employees: 298 nurses, 95 doctors, 86 technical staff, 55 administrators and 43 maintenance staff	576	1.3
	Shidrawi, 2004 (71)	Sana'a	-	Cross-sectional	Health care workers	546	3.5
Special Clinical populations	Gunaid, 1997 (74)	-	-	Cross-sectional	Patients with acute viral hepatitis	78	6.4
	Bakhubaira, 2009 (81)	Aden	2007–2008	Retrospective	Patients with breast cancer, gastrointestinal malignancies and lymphomas	449	8
	Haidar, 2002 (68)	Hajjah	1997–1999	Cross-sectional	Patients suspected to have liver disease	749	8.8
	Salem, 2009 (78)	Sana'a	2005–2007	Case-control	Cases with NHL (females) treated in the haematology and oncology unit (patients coming from different parts of the country)	75	10.7
	Al-Mansoob, 2013 (75)	Sana'a	2009–2011	Cross-sectional	Patients in surgical departments	394	14.2
	Salem, 2009 (78)	Sana'a	2005–2007	Case-control	Cases with NHL (males) treated in the haematology and oncology unit (patients coming from different parts of the country)	117	17.6
	Selm, 2010 (69)	Aden	2007	Cross-sectional	Patients with HCC, CLD, and cirrhosis	67	17.9
	Denis, 1994 (73)	-	1988–1990	Case-control	Cases with leprosy	117	21
	El Guneid, 1993 (80)	Taiz, Sana'a	-	Cross-sectional	Patients with CLD	107	21.8
	Saeed, 2012 (77)	Sana'a	2008–2010	Cross-sectional	Patients with HCC	88	28.4
	Al-Selwi, 2009 (82)	Sana'a	2004–2007	Cross-sectional	Patients with HCC	54	37
	Al-Moslih, 2001 (76)	Sana'a	-	Case-control	Cases with acute and CLD (liver disease, acute viral hepatitis, chronic viral hepatitis, and cryptic or autoimmune disease)	286	37.1
	Salem, 2012 (79)	Sana'a	2001–2008	Cross-sectional	Patients with HCC (with cirrhosis in 187 patients and non-cirrhosis in 64 patients)	251	38.2

*Prevalence figures with more than one decimal place were rounded to one decimal place.

Abbreviations: CLD: chronic liver disease; HCC: hepatocellular carcinoma; NHL: non-Hodgkin lymphomas; STD: sexually transmitted disease; IBBS: integrated bio-behavioural HIV surveillance surveys.

In the general population, rather low HCV prevalence was reported in most studies (n = 9 and median = 1%) [44–49] (Table 1). The lowest HCV prevalence of 0% was reported in a group of children from a government-operated residence for abandoned children [48]; while the highest prevalence of 6.4% was reported in controls in a case-control study [47] (Table 1). The most recent study, conducted in 2011, tested 12,759 blood donors and reported a prevalence of 0.5% [49].

#### Sudan

In high-risk populations, most studies reported high HCV prevalence in the range of 4.5%–34.9% (n = 6 and median = 18.4%) [50–56] (Table 2). High HCV prevalence was reported in clinical populations including HD patients (between 8.5% and 34.9%) [54–56] and haemophilia patients (13%) [53].

In intermediate-risk populations, most studies reported low HCV prevalence in the range of 0%–5.9% (n = 23 and median = 0.5%) [54, 56–60] (Table 2). The majority of HCV prevalence estimates were in MSM and FSWs [57]. The highest prevalence was reported in MSM and FSWs from South Darfur (5.9% and 5.1%, respectively) [57] and in HCWs in a dialysis centre in Khartoum (5.4%) [56].

In special clinical populations, one study reported an HCV prevalence of 11% in patients with HCC [61] and another study reported an HCV prevalence of 6.3% in pregnant women with acute viral hepatitis [62] (Table 2).

In the general population, rather low HCV prevalence was reported in most studies that ranged from 0%-4% (n = 7 and median = 1.3%) [50, 61, 63–67] (Table 1). The lowest prevalence was observed in a study among blood donors at 0% [66] and in a study among pregnant women at 0.6% [63], while the highest prevalence of 4% was reported in non-*Schistosoma-*infected controls [50].

#### Yemen

In high-risk populations, three studies measured HCV prevalence among HD patients and estimated it at a high level of 40% [68], 40.2% [69] and 62.7% [70] (Table 2). In intermediate-risk populations, three studies reported HCV prevalence among HCWs at 0.5% [68], 1.3% [71] and 3.5% [72] (Table 2). In special clinical populations, overall high HCV prevalence was observed in the range of 6.4%–38.2% (n = 13 and median = 17.9%) [68, 70, 73–82] (Table 2). The highest prevalence estimates were reported in patients with acute and chronic hepatitis at 37.1% [76] and in patients with HCC at 38.2% [79].

In the general population, HCV prevalence was in the range of 0%–8.5% (n = 25 and median = 1.3%) [70, 73, 76, 78, 80, 83–92] (Table 1). The highest prevalence at 8.5% was reported in 400 pregnant women in Sana’a in 2011 [85]. The study with the largest sample size (20,329 individuals) reported a prevalence of 4.3% among male controls recruited for a case-control study in 2005–2007.

### Pooled mean HCV prevalence estimates

**Fig 2:**
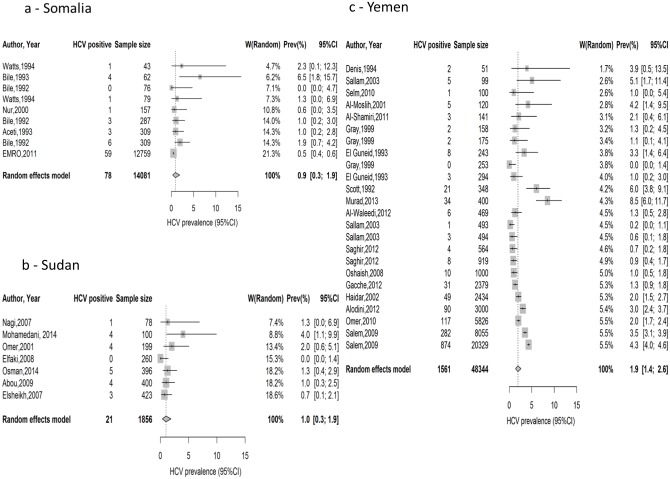
Forest plots presenting the outcomes of the meta-analyses of HCV prevalence studies in the general population in Somalia, Sudan and Yemen.

The pooled country-specific estimates for the national population-level HCV prevalence, based on pooling the general population measures, were: 0.9% (95%CI: 0.3%–1.9%) in Somalia, 1.0% (95%CI: 0.3%–1.9%) in Sudan and 1.9 (95%CI: 1.4%–2.6%) in Yemen. It was also 1.5% (95%CI: 1.0%–2.2%) in this MENA subregion as a whole (Table 3). The corresponding prediction intervals were 0.0%-4.3% in Somalia, 0.0%-4.1% in Sudan, 0.1%-5.7% in Yemen, and 0.0%-7.0% in this MENA subregion as a whole (Table 3). For the meta-analysis in Yemen, we excluded one study presenting HCV prevalence in a migrant non-Yemeni population [84]. Since only one study was conducted in the general population in Djibouti, no meta-analysis was conducted in this country and the estimate in this study of 0.3% (95%CI: 0.1%-0.4%) was taken as the estimate for the national population-level HCV prevalence. The forest plots of the country-specific meta-analyses in the general population can be found in Fig 2.

**Table 3:** Pooled mean HCV prevalence and meta-analysis summary statistics by risk population in Djibouti, Somalia. Sudan and Yemen.

	Number of studies	Total sample size	Prevalence range (%)*	Effect size		Heterogeneity		
				Mean prevalence (%)	Confidence interval (95%)	Q (*p*-value)	I^2^ (confidence interval)	Prediction interval (95%)
Djibouti								
High-risk groups	0	0	-	-	-	-	-	-
Intermediate-risk groups	0	0	-	-	-	-	-	-
Special clinical populations	0	0	-	-	-	-	-	-
General population	1	8,057	0.3	-	0.1–0.4	-	-	-
Somalia								
High-risk groups	0	0	-	-	-	-	-	-
Intermediate-risk groups	5	702	0.0–7.0	1.7	0.0–4.9	17.3 (0.002)	77% (44%-90%)	0.0–18.2
Special clinical populations	2	172	14.5–40.3	-	-	-	-	-
General population	9	14,081	0.0–6.5	0.9	0.3–1.9	24.0 (0.002)	67% (32%-83%)	0.0–4.3
Sudan								
High-risk groups	6	979	4.5–34.8	17.3	8.6–28.2	74.6 (<0.0001)	93.3% (88%-96%)	0.0–60.9
Intermediate-risk groups	23	6,450	0.0–5.4	0.6	0.4–0.8	103.5 (<0.0001)	79% (69%-86%)	0.0–4.3
Special clinical populations	2	131	6.3–11.0	-	-	-	-	-
General population	7	1,856	0.0–4.1	1.0	0.3–1.9	12.9 (0.045)	53% (0%-80%)	0.0–4.1
Yemen								
High-risk groups	3	300	40.0–62.7	-	-	-	-	-
Intermediate-risk groups	3	1,322	0.5–3.5	-	-	-	-	-
Special clinical populations	13	2,832	6.4–38.2	19.4	13.0–26.6	231.8 (<0.001)	95% (93%-96%)	0.9–51.8
General population*	24	48,343	0.0–8.5	1.9	1.4–2.6	345.3 (<0.001)	93% (91%-96%)	0.1–5.7
All countries								
High-risk groups	9	1,279	4.5–62.7	26.2	14.8–39.4	188.3 (<0.0001)	95% (94%-97%)	0.0–76.1
Intermediate-risk groups	31	8,474	0.0–7.0	0.7	0.3–1.3	139.5 (<0.001)	79% (71%-85%)	0.0–4.7
Special clinical populations	17	3,135	6.4–38.2	19.6	13.9–26.0	253.0 (<0.001)	94% (92%-96%)	1.4–50.2
General population*	41	72,337	0.0–8.5	1.5	1.0–2.2	1147.9 (<0.001)	96% (96%-97%)	0.0–7.0

*In the general population of Yemen, one study was not included in the meta-analysis as it reported HCV prevalence in a migrant non-Yemeni population.

Since the number of studies was less than five in some of the risk categories, meta-analyses were not conducted for all risk categories of all countries. Meta-analyses showed a high mean estimate of HCV prevalence in high-risk populations in Sudan at 14.3% (95%CI: 6.2%-24.9%), and in special clinical populations in Yemen at 19.4% (95%CI: 13.9%-26.0%) (Table 3 and S1 Fig). However, the mean estimates of HCV prevalence were low in intermediate-risk populations in Somalia and Sudan, at 1.7% (95%CI: 0.0%-4.9%) and 0.6%, (95%CI: 0.2%-1.1%), respectively (Table 3 and S1 Fig).

There was significant evidence for heterogeneity in effect size in all meta-analyses (Q-test *p*-value <0.10) (Fig 2 and Table 3). I^2^ heterogeneity measure was high in most meta-analyses, and often exceeded 90%, indicating that most of the variation was attributed to variation in effect size across studies rather than chance. The estimated prediction intervals for the pooled means in most analyses were wide suggesting heterogeneity with substantial variation in effect size across individual studies.

### Meta-regression

In the multivariable model adjusting for country, subpopulation within the low risk population, study design, study site, and study precision, the meta-regression did not identify any statistically significant prevalence-modifiable factors apart from country. Both Somalia and Sudan had statistically significantly lower HCV prevalence than Yemen. Relative to Yemen, the prevalence in Somalia was lower with an odds ratio (OR) = 0.44; 95%CI: 0.20–0.96; *p*-value = 0.040. Also relative to Yemen, the prevalence in Sudan was lower with an OR of = 0.39; 95%CI: 0.19–0.82; *p*-value = 0.015. Since there was only one prevalence measure for Djibouti, there was not sufficient statistical power to assess the effect size of this country relative to Yemen.

### Sensitivity analyses

A substantial fraction of HCV prevalence studies contributing to the estimates for the national population-level HCV prevalence were among blood donors (41%). The pooled estimates for the national population-level HCV prevalence, after excluding blood donor data, were 0.9% (95%CI: 0.3%–1.9%) in Somalia, 1.0% (95%CI: 0.3%–1.9%) in Sudan and 1.9% (95%CI: 1.4%–2.6%) in Yemen. There was significant evidence for heterogeneity in effect size in Yemen (p-value <0.001); but the evidence was not significant for Somalia and Sudan.

The pooled estimate in Yemen for the national population-level HCV prevalence, after excluding studies with a sample size lower than 1,000 and published before 2000, was 2.4% (95%CI: 1.6%–3.3%). There was also significant evidence for heterogeneity in effect size (p-value <0.001). This sensitivity analysis was not conducted for Somalia and Sudan. In Somalia, only one study had a sample size greater than 1,000 and was published after 2000 [49]. In Sudan, all studies were published after 2000, but none of them had a sample size greater than 1,000.

### Risk of bias and quality assessment of HCV prevalence studies

This systematic review included only studies in which HCV infection was ascertained using biological assays. This implies that all the included studies have at least one assessment domain at low ROB (S3 and S4 Tables). In the two other assessment domains, proportion of studies with low ROB was 23% for the sampling methodology and 50% for the response rate. Overall, 100% of studies had at least one assessment domain at low ROB, 51% of the studies had at least two assessment domains at low ROB, and 21% of studies had the three assessment domains at low ROB. Meanwhile, 74% of studies had high ROB in at least one assessment domain, 8% of studies had high ROB in at least two assessment domains, and none of the studies had high ROB in all three assessment domains. In terms of precision of estimates, 78% of studies had good precision (S3 and S4 Tables).

### HCV genotypes

This systematic review identified only one and small study describing HCV genotypes [51]. HCV genotype 4 (subtypes e and c/d) was detected in four RNA positive patients out of eight HCV antibody positive cases. These individuals were from a cohort of patients with hepatosplenic schistosomiasis in Khartoum, Sudan.

### Risk factors for HCV infection

Crude (unadjusted) and adjusted risk factors for HCV infection were reported in some of included studies. Multivariable analysis was conducted in only a fraction of studies that reported risk factors. The reported unadjusted risk factors included duration of dialysis [54–56]; history of surgical procedures [54, 75, 85, 86], blood transfusion [75, 83, 85], renal transplantation [54, 65], invasive procedures [75], travel abroad [75], blood donation [83] or jaundice [47]; age [54, 93]; education level [85, 93]; gender [78]; use of khat [47]; level of aminotransferases and alfa-fetoprotein [47] and low parity [85]. The reported adjusted risk factors (after adjustment for confounders) included duration of dialysis [55] and history of blood transfusion [83], blood donation [83] and surgery [86]. Furthermore, HCV infection was significantly associated with HCC [61] and CLD [80] in unadjusted analyses and with NHL [78] in an adjusted analysis.

## Discussion

Building on a developed and refined methodology [7–9, 12–14], we conducted a systematic review and synthesis of HCV prevalence and its epidemiology in Djibouti, Somalia, Sudan, and Yemen. We also presented estimates for the national population-level HCV prevalence and for the prevalence among various at risk categories. The results suggest that national population-level HCV prevalence is at about 1% in Somalia, Sudan, and probably Djibouti, but seems twice as high in Yemen at about 2%. High prevalence estimates were identified in these countries among clinical populations at high risk of infection such as HD and haemophilia patients. Notably, we could not identify any study among PWID in this subregion, a key population at high risk of HCV exposure.

HCV prevalence at the national level in these countries is broadly consistent with the levels observed globally [3, 4] and in the MENA region [7, 10–14]. Our estimates nonetheless contrast with earlier work that suggested higher prevalence in these countries [94, 95]. The estimated infection levels are also substantially lower than those found in Egypt at 14.7% [7, 96] and in Pakistan at 4.7% [97, 98]; the two countries with the highest HCV prevalence in MENA. Yemen appears however to have the third highest HCV prevalence in MENA based on the completed and ongoing analyses of the MENA HCV Epidemiology Synthesis Project [7, 10–14]. This highlights the need to improve our understanding of the epidemiology of HCV in this country, a populous country of 27 million people [99] and ongoing military conflict.

A substantial fraction of HCV prevalence studies in the general population were among blood donors. This population category is biased towards healthy populations and may not necessarily be representative of the wider general population. Accordingly our estimates for HCV prevalence in the general population could underestimate actual prevalence [100, 101]. HCV prevalence could in principle be declining due to population growth combined with a reduction in exposure following the global introduction in the 1990s of more stringent blood screening and infection control protocols. We conducted two sensitivity analyses to examine the impact of excluding blood donors studies and older than 2000 studies on our estimates. The sensitivity analyses indicated a small impact on our estimates for Somalia and Sudan, but suggested that we may be underestimating HCV prevalence in Yemen. Estimated HCV prevalence in Yemen increased from 1.9% in our baseline analysis to 2.8% and 2.4% in the two sensitivity analyses, respectively. These analyses in addition to the meta-regression, therefore affirmed that national population-level HCV prevalence in this subregion of MENA is comparable to global levels, but that Yemen appears to have the third highest HCV prevalence in MENA.

A recent global study by Gower *et al*. has produced an estimate for HCV prevalence in Yemen, but no estimates for HCV prevalence in the other countries of this subregion. The reported estimate by Gower *et al*. is similar to ours (2.2% vs. 1.9%, respectively), however, Gower *et al*. estimate was based on a single study, whereas ours was based on 24 studies [68, 70, 73, 76, 79, 80, 83–93]. The expanded search in our review has allowed us to identify HCV measures published in non-indexed journals and country-level reports. These studies may not have been recognized since they could not be identified using conventional international search engines such as PubMed or Embase. For the same reasons, our study has also identified substantially more HCV data in Somalia and Sudan than recent systematic reviews of HCV prevalence in sub-Saharan Africa [4, 102–104].

Our results suggest ongoing HCV transmission in clinical settings in Djibouti, Somalia, Sudan, and Yemen. This is indicated by the high HCV prevalence in high risk and special clinical populations, regardless of the year of study. Ongoing transmission in clinical settings was also indicated by the significant association between HCV infection and healthcare procedures such as dialysis [55], blood transfusion [83], blood donation [83] and surgery [86]. Ongoing transmission in clinical settings was also suggested by the higher HCV prevalence in intermediate-risk populations, such as hospitalized adults and children and HCWs.

Community transmissions of HCV infection appear to occur in the south-west region of the Arabian Peninsula [83, 105]. In the south-west region of the Kingdom of Saudi Arabia (KSA), the bordering region of Yemen, HCV transmission was linked to traditional phlebotomy (cupping with blood-letting, known in the Arab world as *hijama*) [105]. The south-west region of KSA presented similar HCV prevalence levels with Yemen and the highest HCV prevalence compared with the other regions in KSA [10]. In Yemen, cupping was significantly associated with increased odds of hepatitis B [83], another blood borne virus sharing transmission routes with HCV through infected blood and needles [106, 107]. As it was observed in Egypt [108] and in Japan [109], folk remedies such as traditional phlebotomy using non-sterilized knives should be considered as possible HCV transmission routes potentially explaining the higher HCV prevalence in Yemen and more broadly in the south-west region of the Arabian Peninsula.

PWID [110–112], prisoners [113] and HIV infected MSM [114] are key contributors to HCV transmission dynamics in both developed and developing countries [113, 115]. We could not identify any study of HCV prevalence among PWID, prisoners, or HIV infected MSM; a major shortcoming of the epidemiological evidence in this subregion. The estimated population proportion of injecting drug use is 0.03% in Somalia, 0.20% in Sudan, and 0.23% in Yemen [116]. The population proportion of injecting drug use in MENA is in the intermediate range compared to global levels at 0.24% [113]. The population proportion of MSM in MENA seems to be consistent with reported global levels of 2%–3% [117]. Substantial HIV prevalence has already been identified among MSM in MENA such as in Sudan where HIV prevalence was estimated in different studies at 8–9% [117]. Prison population rates were 83, 13.3, 32.5, 56, and 55 per 100,000 of national populations in Djibouti, Somalia, South Sudan, Sudan, and Yemen in 2013 [118]. These estimates suggest the potential existence of large and hidden high risk populations where the level of infection is unknown, but probably substantial given HCV prevalence levels in these high risk populations in other MENA countries [113, 115, 118, 119] and globally [114, 116, 120]. This lack of evidence for PWID, prisoners, and HIV infected MSM makes it difficult to assess the relative importance of these key risk groups as contributors to HCV transmission dynamics in this subregion.

As discussed in details previously [7–14], among the limitations of such systematic review and meta-analyses is the variability in quantity and quality of studies across countries. For instance, only one study was identified for Djibouti, while 44 studies were identified for Yemen. The pooled mean HCV prevalence estimates may have been also affected by selection bias or investigator bias in included studies. The meta-analyses confirmed considerable heterogeneity in effect size across studies, but the sources of variation remain poorly understood. Given the possibility that this heterogeneity may reflect non-random biases, the prediction intervals calculated for each risk population category (Table 3) may provide a more interpretable summary of the variation in effect size and potential true population mean. We classified the populations into different risk categories based on convention in the literature; however, there is no established existing classification of risk for some populations [12]. We classified these based on our best judgment of HCV risk of exposure.

## Conclusions

The national population-level HCV prevalence is at about 1% in Somalia, Sudan, and probably Djibouti, and is comparable to most countries in MENA and globally. However, HCV prevalence in Yemen appears to be at a higher level, at about 2%, making Yemen the country with the third highest HCV prevalence in MENA based on the MENA HCV Epidemiology Synthesis Project analyses [7, 10–13, 121]. The high HCV prevalence found among different clinical populations suggests ongoing HCV transmission in clinical settings. This points out the need to implement strict measures to prevent HCV transmission such as improved injection safety and properly screened blood transfusions [122, 123]. Establishment of HCV treatment programs should also be explored given the increasing availability and affordability of the new antiviral treatments and generics to cure HCV infection [124–126].

Strikingly, we could not identify any study among PWID, prisoners or HIV infected MSM, a major shortcoming of the epidemiological evidence in this subregion. The contributions of these populations to HCV incidence and prevalence remain unknown. An improved understanding of the epidemiology of HCV infection in this subregion is warranted; this resource-limited subregion remains the least studied subregion in MENA. Improved understanding can be achieved by conducting representative surveys among at risk populations as well as nationally-representative population-based surveys to estimate infection and disease occurrence, geographic variability, modes of exposure, and HCV knowledge and attitudes, as has been done recently in other MENA countries such as Egypt [6, 127–131].

## Supporting Information

S1 Fig Forest plots presenting the outcomes of the meta-analyses of HCV prevalence studies among intermediate risk groups in Somalia and Sudan, special clinical populations groups in Yemen, and high risk groups in Sudan. (TIF)

S1 Table PRISMA 2009 Checklist. (PDF)

S2 Table Search criteria for the systematic review of HCV antibody prevalence in Djibouti, Somalia, Sudan, and Yemen. (PDF)

S3 Table Study-level assessment of precision and risk of bias in HCV antibody prevalence measures, as extracted from eligible reports. (PDF)

S4 Table Summary of study-level assessment of precision and risk of bias in HCV antibody prevalence measures, as extracted from eligible reports. (PDF)

## Abbreviations

95%CI: 95% confidence interval
CLD: chronic liver disease
EMRO: WHO Regional Office for the Eastern Mediterranean
FSW: female sex workers
HCC: hepatocellular carcinoma
HCV: hepatitis C virus
HCWs: healthcare workers
HD: haemodialysis
IAS: International AIDS Society
IBBS: integrated bio-behavioural HIV surveillance surveys
KSA: Kingdom of Saudi Arabia
MENA: Middle East and North Africa
MSM: men who have sex with men
n: number of studies
NHL: non-Hodgkin lymphoma
OR: odds ratio
PRISMA: Preferred Reporting Items for Systematic Reviews and Meta-Analyses
*p*-value: probability value
PWID: people who inject drugs
ROB: risk of bias
SI: supporting information
SOS institution: Société Organisation Sociale institution
STD: sexually transmitted disease
UNAIDS: the Joint United Nations Programme on HIV/AIDS
WHO: World Health Organization

## References

[1] Frank C, Mohamed MK, Strickland GT, Lavanchy D, Arthur RR, Magder LS, et al The role of parenteral antischistosomal therapy in the spread of hepatitis C virus in Egypt. Lancet (London, England). 2000;355(9207):887–91. Epub 2001/02/07. . 10752705 10.1016/s0140-6736(99)06527-7

[2] Strickland GT. Liver disease in Egypt: hepatitis C superseded schistosomiasis as a result of iatrogenic and biological factors. Hepatology (Baltimore, Md). 2006;43(5):915–22. Epub 2006/04/22. 10.1002/hep.21173 . 16628669

[3] Cornberg M, Razavi HA, Alberti A, Bernasconi E, Buti M, Cooper C, et al A systematic review of hepatitis C virus epidemiology in Europe, Canada and Israel. Liver international: official journal of the International Association for the Study of the Liver. 2011;31 Suppl 2:30–60. Epub 2011/06/18. 10.1111/j.1478-3231.2011.02539.x . 21651702

[4] Lavanchy D. Evolving epidemiology of hepatitis C virus. Clinical microbiology and infection: the official publication of the European Society of Clinical Microbiology and Infectious Diseases. 2011;17(2):107–15. Epub 2010/11/26. 10.1111/j.1469-0691.2010.03432.x . 21091831

[5] Mohd Hanafiah K, Groeger J, Flaxman AD, Wiersma ST. Global epidemiology of hepatitis C virus infection: new estimates of age-specific antibody to HCV seroprevalence. Hepatology (Baltimore, Md). 2013;57(4):1333–42. Epub 2012/11/23. 10.1002/hep.26141. . 23172780 10.1002/hep.26141

[6] El-Zanaty F, Way A. Egypt Demographic and Health Survey 2008. Cairo: Egyptian Ministry of Health, National Population Council, El-Zanaty and Associates, and ORC Macro; 2009 431 p.

[7] Mohamoud YA, Mumtaz GR, Riome S, Miller D, Abu-Raddad LJ. The epidemiology of hepatitis C virus in Egypt: a systematic review and data synthesis. BMC infectious diseases. 2013;13:288 10.1186/1471-2334-13-288 23799878 PMC3702438

[8] Chaabna K, Mohamoud YA, Chemaitelly H, Mumtaz GR, Abu-Raddad LJ. Protocol for a systematic review and meta-analysis of hepatitis C virus (HCV) prevalence and incidence in the Horn of Africa sub-region of the Middle East and North Africa. Systematic reviews. 2014;3:146 Epub 2014/12/18. 10.1186/2046-4053-3-146 ; PubMed Central PMCID: PMCPmc4274704. 25516265 PMC4274704

[9] Chaabna K, Mohamoud YA, Chemaitelly H, Mumtaz GR, Abu-Raddad LJ. Protocol for a systematic review and meta-analysis of hepatitis C virus (HCV) prevalence and incidence in The Horn of Africa sub-region of the Middle East and North Africa. 2014. Available at: http://www.crd.york.ac.uk/NIHR_PROSPERO/display_record.asp?ID=CRD42014010318#.VRpKHuEQuCd 2014. 10.1186/2046-4053-3-146 PMC4274704 25516265

[10] Mohamoud YA, Riome S, Abu-Raddad LJ. The epidemiology of hepatitis C virus in the Arabian Gulf countries: Systematic review and meta-analyses (In press). 2015. 10.1016/j.ijid.2016.03.012 26996460

[11] Mahmud S, Akbarzadeh V, Abu-Raddad LJ. The epidemiology of hepatitis C virus in Iran: Systematic review and meta-analyses (In press). 2015. 10.1038/s41598-017-18296-9 PMC5760657 29317673

[12] Fadlalla FA, Mohamoud YA, Mumtaz GR, Abu-Raddad LJ. The epidemiology of hepatitis C virus in the maghreb region: systematic review and meta-analyses. PloS one. 2015;10(3):e0121873 Epub 2015/03/25. 10.1371/journal.pone.0121873 . 25803848 PMC4372394

[13] Chemaitelly H, Chaabna K, Abu-Raddad LJ. The Epidemiology of Hepatitis C Virus in the Fertile Crescent: Systematic Review and Meta-Analysis. PloS one. 2015;10(8):e0135281 Epub 2015/08/22. 10.1371/journal.pone.0135281 . 26296200 PMC4546629

[14] Chemaitelly H, Mahmud S, Rahmani AM, Abu-Raddad LJ. The epidemiology of hepatitis C virus in Afghanistan: Systematic review and meta-analysis. International journal of infectious diseases: IJID: official publication of the International Society for Infectious Diseases. 2015 Epub 2015/09/30. 10.1016/j.ijid.2015.09.011 . 26417880

[15] Moher D, Liberati A, Tetzlaff J, Altman DG. Preferred reporting items for systematic reviews and meta-analyses: the PRISMA statement. Journal of clinical epidemiology. 2009;62(10):1006–12. Epub 2009/07/28. 10.1016/j.jclinepi.2009.06.005 . 19631508

[16] WHO. World Health Organization African Index Medicus (AIM database) [cited 2014]. Available from: http://indexmedicus.afro.who.int/cgi-bin/wxis.exe/iah/?IsisScript=iah/iah.xis&lang=I&base=AIM.

[17] WHO. World Health Organization Index Medicus for the Eastern Mediterranean Region (IMEMR). Available from: http://www.emro.who.int/information-resources/imemr/imemr.html.

[18] Yemeni Journal of Medical Sciences. 2015. Available at: http://ust.edu/ojs/index.php?journal=yjmp 2015. Available from: http://ust.edu/ojs/index.php?journal=yjmp.

[19] Sudan Journal of Medical Sciences. 2015. Available at: http://www.ajol.info/index.php/sjms/index. Available from: http://www.ajol.info/index.php/sjms/index.

[20] Sudan Medical Journal. 2015. Available at: http://www.smj.eg.net/. Available from: http://www.smj.eg.net/.

[21] Abu-Raddad L, Akala FA, Semini I, Riedner G, Wilson D, Tawil O. Characterizing the HIV/AIDS epidemic in the Middle East and North Africa: Time for Strategic Action Middle East and North Africa HIV/AIDS Epidemiology Synthesis Project. World Bank/UNAIDS/WHO Publication. Washington DC: The World Bank Press World Bank/UNAIDS/WHO Publication. Washington DC: The World Bank Press, 2010.

[22] Abu-Raddad LJ, Hilmi N, Mumtaz G, Benkirane M, Akala FA, Riedner G, et al Epidemiology of HIV infection in the Middle East and North Africa. AIDS (London, England). 2010;24 Suppl 2:S5–23. Epub 2010/07/17. . 20610949 10.1097/01.aids.0000386729.56683.33

[23] International AIDS Society conferences. 2015. Available at: https://www.iasociety.org/Default.aspx?pageId=3. Available from: https://www.iasociety.org/Default.aspx?pageId=3.

[24] NIH. Endemic and Emerging Viral Diseases of Priority in the Middle East and North Africa (MENA)—A Scientific Workshop to Promote Research Collaborations, Doha, Qatar. Available at: https://respond.niaid.nih.gov/conferences/qatarmenaworkshop/Pages/default.aspx 2014. Available from: https://respond.niaid.nih.gov/conferences/qatarmenaworkshop/Pages/default.aspx.

[25] Shire AM, Sandhu DS, Kaiya JK, Oseini AM, Yang JD, Chaiteerakij R, et al Viral hepatitis among Somali immigrants in Minnesota: association of hepatitis C with hepatocellular carcinoma. Mayo Clinic proceedings. 2012;87(1):17–24. Epub 2012/01/04. 10.1016/j.mayocp.2011.08.001 ; PubMed Central PMCID: PMCPmc3337857. 22212964 PMC3337857

[26] Endnote X7. Thomson Reuters, San Francisco, CA. Available at: http://endnote.com/downloads.

[27] R v.3.1.1. R Development Core Team, 2011. Available at: http://cran.r-project.org/bin/windows/base/.

[28] Schwarzer G. General Package for Meta-Analysis. Version 4.1–0. Available at: http://cran.r-project.org/web/packages/meta/meta.pdf 2015. Available from: http://cran.r-project.org/web/packages/meta/meta.pdf.

[29] Stata/se 13.1. StataCorp. Stata: Release 13. Statistical Software. College Station, TX: StataCorp LP. 2013.

[30] Clopper CJ, P E.S. The use of confidence or fiducial limits illustrated in the case of the binomial. Biometricka. 1934;26:404–13.

[31] Freeman M, Tukey J. Transformations related to the angular and the square root. The Annals of Mathematical Statistics. 1950;21:607–11.

[32] DerSimonian R, Laird N. Meta-analysis in clinical trials. Controlled clinical trials. 1986;7(3):177–88. Epub 1986/09/01. . 3802833 10.1016/0197-2456(86)90046-2

[33] Miller JJ. The inverse of the Freeman-Tuckey double arcine transformation. The American Statistician. 1978;32(4):138.

[34] Schwarzer G. Meta package. 2014.

[35] Cochran W. The combination of estimates from different experiments. Biometrics. 1954;10(1):101–29.

[36] Higgins J, Thompson S, Deeks J, Altman D. Measuring inconsistency in meta-analyses. BMJ (Clinical research ed). 2003;327:557–60. 10.1136/bmj.327.7414.557 PMC192859 12958120

[37] Riley RD, Higgins JP, Deeks JJ. Interpretation of random effects meta-analyses. BMJ (Clinical research ed). 2011;342:d549 Epub 2011/02/12. 10.1136/bmj.d549 . 21310794

[38] Borenstein M. Introduction to meta-analysis. Chichester, U.K.: John Wiley & Sons; 2009 xxviii, 421 p. p.

[39] Higgins JP, Thompson SG. Quantifying heterogeneity in a meta-analysis. Statistics in medicine. 2002;21(11):1539–58. Epub 2002/07/12. 10.1002/sim.1186 . 12111919

[40] Higgins JPT, Green S. Cochrane Handbook for Systematic Reviews of Interventions Collaboration TC, editor2008.

[41] Gower E, Estes C, Blach S, Razavi-Shearer K, Razavi H. Global epidemiology and genotype distribution of the hepatitis C virus infection. J Hepatol. 2014;61(1 Suppl):S45–57. 10.1016/j.jhep.2014.07.027 . 25086286

[42] Galea S, Tracy M. Participation rates in epidemiologic studies. Annals of epidemiology. 2007;17(9):643–53. Epub 2007/06/08. 10.1016/j.annepidem.2007.03.013 . 17553702

[43] Dray X, Dray-Spira R, Bronstein JA, Mattera D. [Prevalences of HIV, hepatitis B and hepatitis C in blood donors in the Republic of Djibouti]. Med Trop (Mars). 2005;65(1):39–42. Epub 2005/05/21. . 15903075

[44] Aceti A, Taliani G, Bruni R, Sharif OS, Moallin KA, Celestino D, et al Hepatitis C virus infection in chronic liver disease in Somalia. Am J Trop Med Hyg. 1993;48(4):581–4. Epub 1993/04/01. . 7683179 10.4269/ajtmh.1993.48.581

[45] Nur YA, Groen J, Elmi AM, Ott A, Osterhaus AD. Prevalence of serum antibodies against bloodborne and sexually transmitted agents in selected groups in Somalia. Epidemiology and infection. 2000;124(1):137–41. Epub 2000/03/18. ; PubMed Central PMCID: PMCPmc2810894. 10722141 10.1017/s0950268899003441 PMC2810894

[46] Watts DM, Corwin AL, Omar MA, Hyams KC. Low risk of sexual transmission of hepatitis C virus in Somalia. Transactions of the Royal Society of Tropical Medicine and Hygiene. 1994;88(1):55–6. Epub 1994/01/01. . 8154002 10.1016/0035-9203(94)90495-2

[47] Bile K, Aden C, Norder H, Magnius L, Lindberg G, Nilsson L. Important role of hepatitis C virus infection as a cause of chronic liver disease in Somalia. Scand J Infect Dis. 1993;25(5):559–64. Epub 1993/01/01. . 7506842 10.3109/00365549309008543

[48] Bile K, Mohamud O, Aden C, Isse A, Norder H, Nilsson L, et al The risk for hepatitis A, B, and C at two institutions for children in Somalia with different socioeconomic conditions. Am J Trop Med Hyg. 1992;47(3):357–64. Epub 1992/09/01. . 1524149 10.4269/ajtmh.1992.47.357

[49] WHO. EMRO Annual HIV STI reporting form SOMALIA 2011—Hepatitis C. 2011.

[50] Mohamedani A. Prevalence of Hepatitis C Virus Antibodies in Patients with Schistosomiasis in Gezira State in Central Sudan. Endemic and Emerging Viral Diseases of Priority in the Middle East and North Africa (MENA)—A Scientific Workshop to Promote Research Collaboration; May 2014; Doha, Qatar2014. p. 86.

[51] Mudawi HM, Smith HM, Fletcher IA, Fedail SS. Prevalence and common genotypes of HCV infection in Sudanese patients with hepatosplenic schistosomiasis. Journal of medical virology. 2007;79(9):1322–4. Epub 2007/07/04. 10.1002/jmv.20865 . 17607776

[52] Mudawi HM, Smith HM, Rahoud SA, Fletcher IA, Babikir AM, Saeed OK, et al Epidemiology of HCV infection in Gezira state of central Sudan. Journal of medical virology. 2007;79(4):383–5. Epub 2007/02/22. 10.1002/jmv.20780 . 17311341

[53] Gadour MOEH, Mohamed BT. HBV, HCV and HIV among patients with Hemophilia in Khartoum—Sudan. Sudan Journal of Medical Sciences. 2011;6(4):233–7.

[54] El-Amin HH, Osman EM, Mekki MO, Abdelraheem MB, Ismail MO, Yousif ME, et al Hepatitis C virus infection in hemodialysis patients in Sudan: two centers' report. Saudi journal of kidney diseases and transplantation: an official publication of the Saudi Center for Organ Transplantation, Saudi Arabia. 2007;18(1):101–6. Epub 2007/01/24. . 17237901

[55] Gasim GI, Hamdan HZ, Hamdan SZ, Adam I. Epidemiology of hepatitis B and hepatitis C virus infections among hemodialysis patients in Khartoum, Sudan. Journal of medical virology. 2012;84(1):52–5. Epub 2011/11/05. 10.1002/jmv.22256 . 22052648

[56] Suliman SM, Fessaha S, El Sadig M, El-Hadi MB, Lambert S, Fields H, et al Prevalence of hepatitis C virus infection in hemodialysis patients in Sudan. Saudi journal of kidney diseases and transplantation: an official publication of the Saudi Center for Organ Transplantation, Saudi Arabia. 1995;6(2):154–6. Epub 1995/04/01. . 18583856

[57] IBBS National Team. Integrated bio-behavioral HIV surveillance (IBBS) among female sex workers and men who have sex with men in 15 states of Sudan, 2011–2012. 2013.

[58] Nail A, Eltiganni S, Imam A. Seroprevalence of Hepatitis B and C among health care workers in Omdurman, Sudan. Sudan Journal of Medical Sciences. 2008;3(3):201–5.

[59] Mudawi H, Hussein W, Mukhtar M, Yousif M, Nemeri O, Glebe D, et al Overt and occult hepatitis B virus infection in adult Sudanese HIV patients. International journal of infectious diseases: IJID: official publication of the International Society for Infectious Diseases. 2014;29:65–70. Epub 2014/12/03. 10.1016/j.ijid.2014.07.004 . 25449238

[60] McCarthy MC, el-Tigani A, Khalid IO, Hyams KC. Hepatitis B and C in Juba, southern Sudan: results of a serosurvey. Transactions of the Royal Society of Tropical Medicine and Hygiene. 1994;88(5):534–6. Epub 1994/09/01. . 7992329 10.1016/0035-9203(94)90150-3

[61] Omer RE, Van't Veer P, Kadaru AM, Kampman E, el Khidir IM, Fedail SS, et al The role of hepatitis B and hepatitis C viral infections in the incidence of hepatocellular carcinoma in Sudan. Transactions of the Royal Society of Tropical Medicine and Hygiene. 2001;95(5):487–91. Epub 2001/11/15. . 11706655 10.1016/s0035-9203(01)90013-6

[62] Ahmed RE, Karsany MS, Adam I. Brief report: acute viral hepatitis and poor maternal and perinatal outcomes in pregnant Sudanese women. Journal of medical virology. 2008;80(10):1747–8. Epub 2008/08/21. 10.1002/jmv.21284 . 18712815

[63] Elsheikh RM, Daak AA, Elsheikh MA, Karsany MS, Adam I. Hepatitis B virus and hepatitis C virus in pregnant Sudanese women. Virol J. 2007;4:104 Epub 2007/10/26. 10.1186/1743-422x-4-104 ; PubMed Central PMCID: PMCPmc2116999. 17958904 PMC2116999

[64] Abou MA, Eltahir YM. Seropositivity of hepatitis B virus and hepatitis C virus dual infection among blood donors in Nyala teaching hospital. Virol J. 2009;6:227 Epub 2009/12/24. 10.1186/1743-422x-6-227 ; PubMed Central PMCID: PMCPmc2804613. 20028507 PMC2804613

[65] Nagi AM, Al Tayeb HA, Ahmed MA. Seroprevalence of hepatitis Band C viral infections among blood donors in Shendi, River Nile State, Sudan. Research Journal of Medicine and Medical Sciences. 2007;2(2):122–6.

[66] Elfaki AMH, Eldour AAA, Elsheikh NMH. Sero-prevalence of immunodeficiency virus, hepatitis B and C and syphilis among blood donors at ElObeid Teaching Hospital, West Sudan. Sudan Journal of Medical Sciences. 2008;3(4):333–7.

[67] Osman AMM, Minrghani OA, Gasim GI, Adam I. Hepatitis B Virus, Hepatitis C Virus and Human Immunodeficiency Virus Infection among Pregnant Women in Central Sudan. Sudanese Journal of Medical Sciences. 2014;9(2):91–5.

[68] Haidar NA. Prevalence of hepatitis B and hepatitis C in blood donors and high risk groups in Hajjah, Yemen Republic. Saudi Med J. 2002;23(9):1090–4. Epub 2002/10/09. . 12370719

[69] Aman K, Al-Dubai SA, Aman R, Hawash A, Alshagga M, Kassim S. Prevalence and associated factors of hepatitis C virus infection among renal disease patients on maintenance hemodialysis in three health centers in Aden, Yemen: a cross sectional study. Saudi journal of kidney diseases and transplantation: an official publication of the Saudi Center for Organ Transplantation, Saudi Arabia. 2015;26(2):380–5. Epub 2015/03/12. . 25758898 10.4103/1319-2442.152555

[70] Selm SB. Prevalence of hepatitis C virus infection among hemodialysis patients in a single center in Yemen. Saudi journal of kidney diseases and transplantation: an official publication of the Saudi Center for Organ Transplantation, Saudi Arabia. 2010;21(6):1165–8. Epub 2010/11/10. . 21060200

[71] Al-Jarba AS, Al-Sayyari WM. Prevalence of hepatitis B virus and hepatitis C virus in health workers in 3 major hospitals in Aden, Republic of Yemen. Saudi Med J. 2003;24(9):1031–2. Epub 2003/09/16. . 12973499

[72] Shidrawi R, Ali Al-Huraibi M, Ahmad Al-Haimi M, Dayton R, Murray-Lyon IM. Seroprevalence of markers of viral hepatitis in Yemeni healthcare workers. Journal of medical virology. 2004;73(4):562–5. Epub 2004/06/29. 10.1002/jmv.20126 . 15221900

[73] Denis F, Aussel L, Ranger S, Martin P, Itoua-N'Gaporo A, Frommel D, et al Prevalence of antibodies to hepatitis C virus among patients with leprosy in several African countries and the Yemen. Journal of medical virology. 1994;43(1):1–4. Epub 1994/05/01. . 7521898 10.1002/jmv.1890430102

[74] Gunaid AA, Nasher TM, el-Guneid AM, Hill M, Dayton R, Pal A, et al Acute sporadic hepatitis in the Republic of Yemen. Journal of medical virology. 1997;51(1):64–6. Epub 1997/01/01. . 8986951 10.1002/(sici)1096-9071(199701)51:1<64::aid-jmv10>3.0.co;2-4

[75] Al-Mansoob AS, Salem AK, A-Selwi A.H.A., Assamawi A. Risk factors of hepatitis B and C viruses among patients admitted in surgical departments at Al-Thawra Hospital, Sana'a, Yemen. Sudan Medical Journal. 2013;49(3):168–75. Epub December 2013.

[76] Al-Moslih MI, Al-Huraibi MA. Prevalence of hepatitis C virus among patients with liver disease in the Republic of Yemen. East Mediterr Health J. 2001;7(4–5):771–8. Epub 2004/08/31. . 15332778

[77] Saeed NM, Bawazir AA, Al-Zuraiqi M, Al-Negri F, Yunus F. Why is hepatocellular carcinoma less attributable to viral hepatitis in Yemen? Asian Pacific journal of cancer prevention: APJCP. 2012;13(8):3663–7. Epub 2012/10/27. . 23098451 10.7314/apjcp.2012.13.8.3663

[78] Salem AK. Prevalence of HCV among Yemeni patients with non-Hodgkin's lymphoma at AI-Thawra teaching hospital. Gulf J Oncolog. 2009;(5):22–9. Epub 2010/01/21. . 20084782

[79] Salem AK, Abdulrab A, Alfakeh Y, Aown A. Hepatocellular carcinoma in Yemeni patients: a single centre experience over an 8-year period. East Mediterr Health J. 2012;18(7):693–9. Epub 2012/08/16. . 22891515 10.26719/2012.18.7.693

[80] El Guneid AM, Gunaid AA, O'Neill AM, Zureikat NI, Coleman JC, Murray-Lyon IM. Prevalence of hepatitis B, C, and D virus markers in Yemeni patients with chronic liver disease. Journal of medical virology. 1993;40(4):330–3. Epub 1993/08/01. . 8228926 10.1002/jmv.1890400413

[81] Bakhubaira S. Hepatitis B, C and human immune deficiency virus among cancer patients attending the National Cancer of Public Health Laboratories—Aden. Abstracts of Researches. 2009;13(2).

[82] Al-Selwi AHA, Elezzy Y, Al Ghazali J, Hadi S. Association of hepatocellular carcinoma with hepatic viral markers B and C among Yemenis patients at Althawra Hospital Sana'a. Sudan Medical Journal. 2009;4(3):237–42.

[83] Al-Waleedi AA, Khader YS. Prevalence of hepatitis B and C infections and associated factors among blood donors in Aden City, Yemen. East Mediterr Health J. 2012;18(6):624–9. Epub 2012/08/15. . 22888620 10.26719/2012.18.6.624

[84] Sallam TA, Tong CY, Cuevas LE, Raja'a YA, Othman AM, Al-Kharsa KR. Prevalence of blood-borne viral hepatitis in different communities in Yemen. Epidemiology and infection. 2003;131(1):771–5. Epub 2003/09/02. ; PubMed Central PMCID: PMCPmc2870019. 12948378 10.1017/s0950268803008653 PMC2870019

[85] Murad EA, Babiker SM, Gasim GI, Rayis DA, Adam I. Epidemiology of hepatitis B and hepatitis C virus infections in pregnant women in Sana'a, Yemen. BMC Pregnancy Childbirth. 2013;13:127 Epub 2013/06/14. 10.1186/1471-2393-13-127 ; PubMed Central PMCID: PMCPmc3684507. 23758990 PMC3684507

[86] Scott DA, Constantine NT, Callahan J, Burans JP, Olson JG, al-Fadeel M, et al The epidemiology of hepatitis C virus antibody in Yemen. Am J Trop Med Hyg. 1992;46(1):63–8. Epub 1992/01/01. . 1311155 10.4269/ajtmh.1992.46.63

[87] Al-Shamiri AH, Al-Taj MA, Ahmed AS. Prevalence and co-infections of schistosomiasis/hepatitis B and C viruses among school children in an endemic areas in Taiz, Yemen. Asian Pac J Trop Med. 2011;4(5):404–8. Epub 2011/07/21. 10.1016/s1995-7645(11)60113-2 . 21771686

[88] Omer HH. Distribution of Hepatitis B virus, and Hepatitis C virus among blood donors in Aden Blood Center. Abstracts of Researches. 2010;14(1):7.

[89] Gray GC, Kassira EN, Rodier GR, Myers MC, Calamaio CA, Gregory M, et al Remote village survey for agents causing hepatosplenic disease in the Republic of Yemen. Tropical doctor. 1999;29(4):212–9. Epub 1999/12/01. . 10578634 10.1177/004947559902900408

[90] Alodini A. Prevalence of Hepatitis B Virus (HBV) and Hepatitis C Virus (HCV) Infection among Blood Donors at Al-Thawra Hospital Sana'a City—Yemen. Yemeni Journal for Medical Sciences. 2012;6:16–20.

[91] Oshaish HA, El Shazly H, Elabsii AR. Prevalence of HBS Ag virus, anti-hepatitis C virus and anti-HIV among volunteer blood donor in Taiz private hospital, Yemen Republic. Assiut Medical Journal. 2008;32(3):163–70.

[92] Saghir SAM, Al-Hassan FM, Alsalahi OSA, Alhariry AEAA, Baqir HS. Frequencies of HBV, HCV, HIV, and syphilis markers among blood donors: A hospital-based study in Hodeidah, Yemen. Tropical Journal of Pharmaceutical Research. 2012;11(1):132–6. 10.4314/tjpr.v11i1.17.

[93] Gacche RN, Al-Mohani SK. Seroprevalence and Risk Factors for Hepatitis C Virus Infection among General Population in Central Region of Yemen. Hepat Res Treat. 2012;2012:689726 Epub 2013/01/16. 10.1155/2012/689726 ; PubMed Central PMCID: PMCPmc3536035. 23320156 PMC3536035

[94] Daw MA, Dau AA. Hepatitis C virus in Arab world: a state of concern. TheScientificWorldJournal. 2012;2012:719494 Epub 2012/05/26. 10.1100/2012/719494 ; PubMed Central PMCID: PMCPmc3354686. 22629189 PMC3354686

[95] Mudawi HM. Epidemiology of viral hepatitis in Sudan. Clinical and experimental gastroenterology. 2008;1:9–13. Epub 2008/01/01. ; PubMed Central PMCID: PMCPmc3108625. 21677820 10.2147/ceg.s3887 PMC3108625

[96] El-Zanaty F, Way A. Egypt Demographic and Health Survey 2008. Cairo, Egypt: Ministry of Health, El-Zanaty and Associates, and Macro International, 2009.

[97] Ali SA, Donahue RM, Qureshi H, Vermund SH. Hepatitis B and hepatitis C in Pakistan: prevalence and risk factors. International journal of infectious diseases: IJID: official publication of the International Society for Infectious Diseases. 2009;13(1):9–19. Epub 2008/10/07. 10.1016/j.ijid.2008.06.019 ; PubMed Central PMCID: PMCPmc2651958. 18835208 PMC2651958

[98] Umar M, Bushra HT, Ahmad M, Data A, Ahmad M, Khurram M, et al Hepatitis C in Pakistan: a review of available data. Hepatitis monthly. 2010;10(3):205–14. Epub 2010/07/01. ; PubMed Central PMCID: PMCPmc3269085. 22308140 PMC3269085

[99] The World Factbook. 2015. Available at: https://www.cia.gov/library/publications/the-world-factbook/.

[100] Zou S, Dorsey KA, Notari EP, Foster GA, Krysztof DE, Musavi F, et al Prevalence, incidence, and residual risk of human immunodeficiency virus and hepatitis C virus infections among United States blood donors since the introduction of nucleic acid testing. Transfusion. 2010;50(7):1495–504. 10.1111/j.1537-2995.2010.02622.x . 20345570

[101] Denniston MM, Jiles RB, Drobeniuc J, Klevens RM, Ward JW, McQuillan GM, et al Chronic hepatitis C virus infection in the United States, National Health and Nutrition Examination Survey 2003 to 2010. Annals of internal medicine. 2014;160(5):293–300. 10.7326/M13-1133 . 24737271 PMC4562398

[102] Rao VB, Johari N, du Cros P, Messina J, Ford N, Cooke GS. Hepatitis C seroprevalence and HIV co-infection in sub-Saharan Africa: a systematic review and meta-analysis. The Lancet Infectious diseases. 2015 Epub 2015/05/10. 10.1016/s1473-3099(15)00006-7 . 25957078

[103] Madhava V, Burgess C, Drucker E. Epidemiology of chronic hepatitis C virus infection in sub-Saharan Africa. The Lancet Infectious diseases. 2002;2(5):293–302. Epub 2002/06/14. . 12062995 10.1016/s1473-3099(02)00264-5

[104] Riou J, Ait Ahmed M, Blake A, Vozlinsky S, Brichler S, Eholie S, et al Hepatitis C virus seroprevalence in adults in Africa: a systematic review and meta-analysis. Journal of viral hepatitis. 2015 Epub 2015/10/20. 10.1111/jvh.12481 . 26477881

[105] Arya SC. Risk factors for acquisition of hepatitis C virus infection in Saudi Arabia. Annals of Saudi medicine. 1996;16(2):229 Epub 1996/03/01. . 17372491

[106] WHO. Hepatitis C. 2015 Availables at: http://www.who.int/mediacentre/factsheets/fs164/en/.

[107] WHO. Hepatitis B. 2015 Availables at: http://www.who.int/mediacentre/factsheets/fs204/en/.

[108] Abd El-Wahab E, Mikheal A, Sidkey F, Shatat HZ. Factors Associated with Hepatitis C Infection among Chronic HCV Egyptian Patients. Iranian journal of public health. 2014;43(11):1510–8. Epub 2015/06/11. ; PubMed Central PMCID: PMCPmc4449500. 26060718 PMC4449500

[109] Kiyosawa K, Tanaka E, Sodeyama T, Yoshizawa K, Yabu K, Furuta K, et al Transmission of hepatitis C in an isolated area in Japan: community-acquired infection. The South Kiso Hepatitis Study Group. Gastroenterology. 1994;106(6):1596–602. Epub 1994/06/01. . 8194707 10.1016/0016-5085(94)90416-2

[110] Alter MJ. Epidemiology of hepatitis C virus infection. World journal of gastroenterology: WJG. 2007;13(17):2436–41. 17552026 10.3748/wjg.v13.i17.2436 PMC4146761

[111] Shepard CW, Finelli L, Alter MJ. Global epidemiology of hepatitis C virus infection. The Lancet Infectious diseases. 2005;5(9):558–67. 10.1016/S1473-3099(05)70216-4 . 16122679

[112] Armstrong GL, Wasley A, Simard EP, McQuillan GM, Kuhnert WL, Alter MJ. The prevalence of hepatitis C virus infection in the United States, 1999 through 2002. Annals of internal medicine. 2006;144(10):705–14. . 16702586 10.7326/0003-4819-144-10-200605160-00004

[113] Mumtaz GR, Weiss HA, Thomas SL, Riome S, Setayesh H, Riedner G, et al HIV among people who inject drugs in the Middle East and North Africa: systematic review and data synthesis. PLoS medicine. 2014;11(6):e1001663 10.1371/journal.pmed.1001663 24937136 PMC4061009

[114] Bradshaw D, Matthews G, Danta M. Sexually transmitted hepatitis C infection: the new epidemic in MSM? Current opinion in infectious diseases. 2013;26(1):66–72. Epub 2012/12/18. . 23242342 10.1097/QCO.0b013e32835c2120

[115] Mumtaz GR, Weiss HA, Abu-Raddad LJ. Hepatitis C virus and HIV infections among people who inject drugs in the Middle East and North Africa: a neglected public health burden? Journal of the International AIDS Society. 2015;18(1):20582 Epub 2015/07/30. 10.7448/ias.18.1.20582 ; PubMed Central PMCID: PMCPmc4518656. 26221872 PMC4518656

[116] Nelson PK, Mathers BM, Cowie B, Hagan H, Des Jarlais D, Horyniak D, et al Global epidemiology of hepatitis B and hepatitis C in people who inject drugs: results of systematic reviews. Lancet (London, England). 2011;378(9791):571–83. Epub 2011/08/02. 10.1016/s0140-6736(11)61097-0 ; PubMed Central PMCID: PMCPmc3285467. 21802134 PMC3285467

[117] Mumtaz G, Hilmi N, McFarland W, Kaplan RL, Akala FA, Semini I, et al Are HIV epidemics among men who have sex with men emerging in the Middle East and North Africa?: a systematic review and data synthesis. PLoS medicine. 2010;8(8):e1000444 Epub 2011/08/11. 10.1371/journal.pmed.1000444 ; PubMed Central PMCID: PMCPmc3149074. 21829329 PMC3149074

[118] Heijnen M, Mumtaz G, Abu-Raddad LJ. Status of HIV and hepatitis C virus infections amongst prisoners in the Middle East and North Africa. (Under preparation). 10.7448/IAS.19.1.20873 PMC4884676 27237131

[119] Mohamed HI, Saad ZM, Abd-Elreheem EM, Abd-ElGhany WM, Mohamed MS, Abd Elnaeem EA, et al Hepatitis C, hepatitis B and HIV infection among Egyptian prisoners: seroprevalence, risk factors and related chronic liver diseases. Journal of infection and public health. 2013;6(3):186–95. Epub 2013/05/15. 10.1016/j.jiph.2012.12.003 . 23668463

[120] Larney S, Kopinski H, Beckwith CG, Zaller ND, Jarlais DD, Hagan H, et al Incidence and prevalence of hepatitis C in prisons and other closed settings: results of a systematic review and meta-analysis. Hepatology (Baltimore, Md). 2013;58(4):1215–24. Epub 2013/03/19. 10.1002/hep.26387 ; PubMed Central PMCID: PMCPmc3723697. 23504650 PMC3723697

[121] Chemaitelly H, Mahmud S, Abu-Raddad LJ. The epidemiology of hepatitis C virus in Afghanistan (under review). 2015. 10.1016/j.ijid.2015.09.011 26417880

[122] WHO. WHO calls for worldwide use of "smart" syringes. 2015. Available at: http://www.who.int/mediacentre/news/releases/2015/injection-safety/en/.

[123] WHO. WHO guideline on the use of safety-engineered syringes for intramuscular, intradermal and subcutaneous injections in health-care settings. Available at: http://www.who.int/injection_safety/global-campaign/injection-safety_guidline.pdf. Geneva, Swizerland: World Health Organization 2015. 26203487

[124] Laboratory. G. Gilead Press Release: U.S. Food and Drug Administration Approves Gilead’s Sovaldi^™^ (Sofosbuvir) for the Treatment of Chronic Hepatitis C. Available at: http://www.gilead.com/news/press-releases/2013/12/us-food-and-drug-administration-approves-gileads-sovaldi-sofosbuvir-for-the-treatment-of-chronic-hepatitis-c 2013. Available from: http://www.gilead.com/news/press-releases/2013/12/us-food-and-drug-administration-approves-gileads-sovaldi-sofosbuvir-for-the-treatment-of-chronic-hepatitis-c.

[125] Cousien A, Tran VC, Deuffic-Burban S, Jauffret-Roustide M, Dhersin JS, Yazdanpanah Y. Hepatitis c treatment as prevention of viral transmission and liver-related morbidity in persons who inject drugs. Hepatology (Baltimore, Md). 2015 Epub 2015/09/22. 10.1002/hep.28227 . 26390137

[126] Martin NK, Vickerman P, Dore GJ, Hickman M. The hepatitis C virus epidemics in key populations (including people who inject drugs, prisoners and MSM): the use of direct-acting antivirals as treatment for prevention. Current opinion in HIV and AIDS. 2015;10(5):374–80. Epub 2015/08/08. . 26248124 10.1097/COH.0000000000000179 PMC4659815

[127] Cuadros DF, Branscum AJ, Miller FD, Abu-Raddad LJ. Spatial epidemiology of hepatitis C virus infection in Egypt: analyses and implications. Hepatology (Baltimore, Md). 2014;60(4):1150–9. Epub 2014/06/11. 10.1002/hep.27248 ; PubMed Central PMCID: PMCPmc4282472. 24913187 PMC4282472

[128] Miller FD, Abu-Raddad LJ. Evidence of intense ongoing endemic transmission of hepatitis C virus in Egypt. Proceedings of the National Academy of Sciences of the United States of America. 2010;107(33):14757–62. Epub 2010/08/11. 10.1073/pnas.1008877107 ; PubMed Central PMCID: PMCPmc2930444. 20696911 PMC2930444

[129] Chemaitelly H, Abu-Raddad LJ, Miller FD. An apparent lack of epidemiologic association between hepatitis C virus knowledge and the prevalence of hepatitis C infection in a national survey in Egypt. PloS one. 2013;8(7):e69803 Epub 2013/08/08. 10.1371/journal.pone.0069803 ; PubMed Central PMCID: PMCPmc3726777. 23922806 PMC3726777

[130] Benova L, Awad SF, Miller FD, Abu-Raddad LJ. Estimation of hepatitis C virus infections resulting from vertical transmission in Egypt. Hepatology. 2015;61(3):834–42. 10.1002/hep.27596 25366418 PMC4365684

[131] Guerra J, Garenne M, Mohamed MK, Fontanet A. HCV burden of infection in Egypt: results from a nationwide survey. Journal of viral hepatitis. 2012;19(8):560–7. 10.1111/j.1365-2893.2011.01576.x . 22762140

