# Supplementary figures and images for: Hepatitis C Virus Epidemiology in Djibouti, Somalia, Sudan, and Yemen: Systematic Review and Meta-Analysis
Source: PLoS One. 2016 Feb 22;11(2):e0149966. doi: 10.1371/journal.pone.0149966 (PMC4764686; doi:10.1371/journal.pone.0149966)

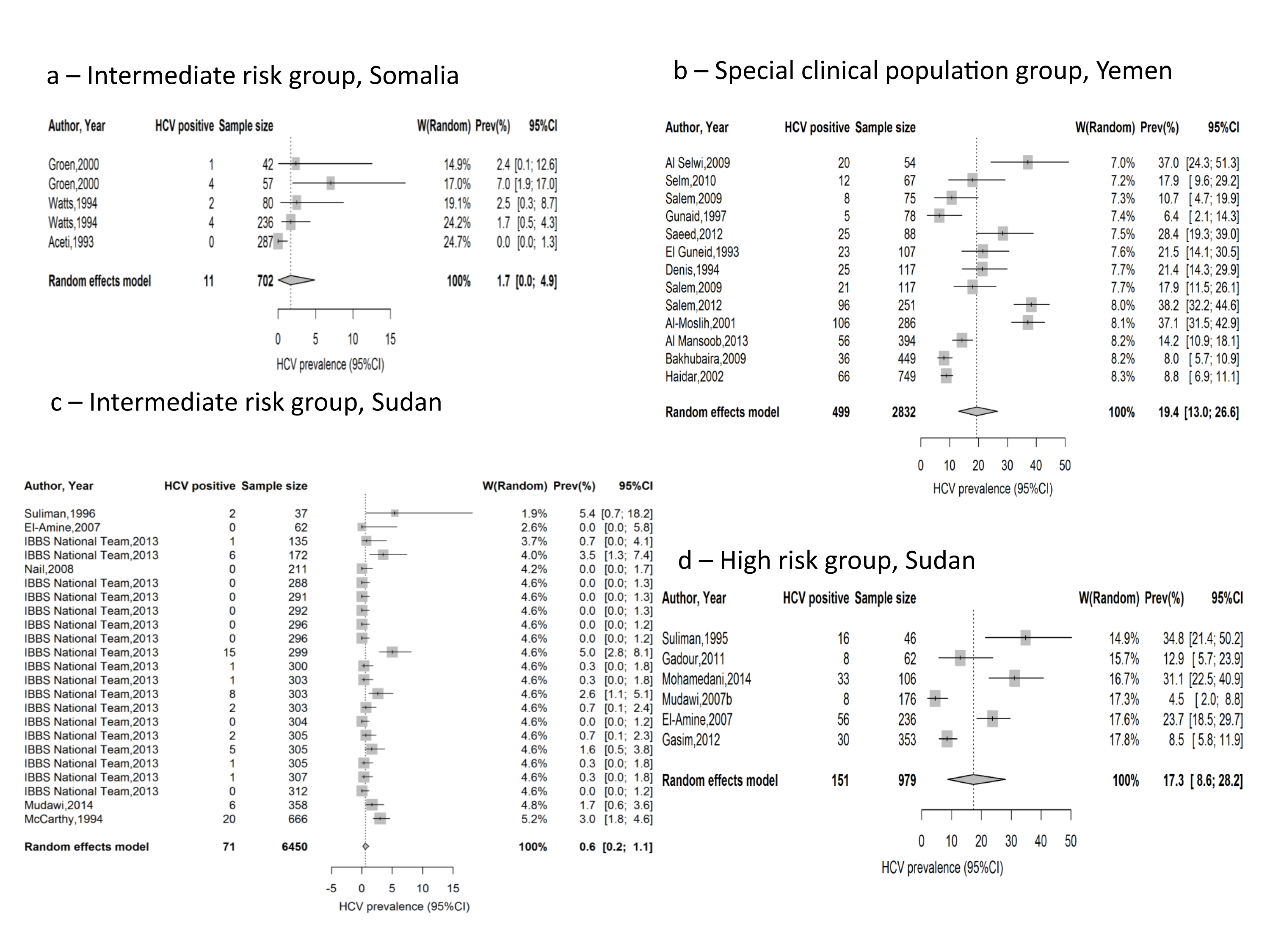

Supplement: S1 Fig — (TIF) [file pone.0149966.s001.tif]
