# Supplementary material for: Hepatitis C Virus Epidemiology in Djibouti, Somalia, Sudan, and Yemen: Systematic Review and Meta-Analysis
Source: PLoS One. 2016 Feb 22;11(2):e0149966. doi: 10.1371/journal.pone.0149966 (PMC4764686; doi:10.1371/journal.pone.0149966)
Supplement: S2 Table — (PDF) [file pone.0149966.s003.pdf]

S2 Table. Search criteria for the systematic review of HCV antibody prevalence in Djibouti, Somalia, Sudan, and Yemen.

PubMed (last searched: May 17, 2015)

("Hepatitis C"[Mesh] OR "Hepatitis C Antibodies"[Mesh] OR "Hepatitis C Antigens"[Mesh] OR "Hepacivirus"[Mesh] OR "Hepatitis C, chronic/epidemiology"[Mesh] OR "Hepatitis C, chronic/etiology"[Mesh] OR "Hepatitis C, chronic/transmission"[Mesh] OR "Hepatitis C, chronic/virology"[Mesh] OR "Hepatitis C"[Text] OR "HCV"[Text] OR "Hepatite"[Text] OR "VHC"[Text] OR "HVC"[Text]) AND ("Yemen"[Mesh] OR "Djibouti"[Mesh] OR "Somalia"[Mesh] OR "Sudan"[Mesh] OR "Africa"[Mesh] OR "Yemen"[Text] OR “Yemeni” [Text] OR "Djibouti"[Text] OR "Somalia"[Text] OR “Somali” [Text] OR "Sudan"[Text] OR “Sudanese” [Text] OR " Africa"[Text] OR “African” [Text])

Embase (last searched: May 17, 2015)

(Yemen*.mp. or exp Yemen/ or Djibouti.mp. or exp Djibouti/ or Somali*.mp. or exp Somalia/ or Sudan*.mp. or exp Sudan/ or Africa*.mp. or exp Africa/) AND (exp hepatitis C/ or exp Hepatitis C virus/ or hepatitis C.mp. or HCV.mp. or hepacivirus.mp. or Hepatite.mp. or VHC.mp. or HVC.mp.)

Regional databases

1. **WHO African Index Medicus (last searched: May 17, 2015)**

Djibouti; Somalia; Sudan; Yemen

2. **Index Medicus for the Eastern Mediterranean Region (last searched: May 17, 2015)**

Djibouti; Somalia; Sudan; Yemen

3. **Middle East and North Africa HIV/AIDS Epidemiology Synthesis Project database (last searched: April 14, 2015)**

All country-level and international organizations’ reports for Djibouti, Somalia, Sudan, and Yemen available in the database.

Abstract archives of the International AIDS Society conferences (last searched: May 17, 2015)

Djibouti; Somalia; Sudan; Yemen
