# Supplementary material for: Hepatitis C Virus Epidemiology in Djibouti, Somalia, Sudan, and Yemen: Systematic Review and Meta-Analysis
Source: PLoS One. 2016 Feb 22;11(2):e0149966. doi: 10.1371/journal.pone.0149966 (PMC4764686; doi:10.1371/journal.pone.0149966)
Supplement: S3 Table — (PDF) [file pone.0149966.s004.pdf]

S3 Table. Study-level assessment of precision and risk of bias in HCV antibody prevalence measures, as extracted from eligible reports.

Author, Year	Study period	Prevalence (%)	Samples tested for HCV	Sampling	Precision	Risk of bias		
						HCV ascertainment	Sampling	Response rate
**Djibouti**								
Dray,2005 [1]	1998-2000	0.3	8,057	Convenience	Good precision	Low ROB	High ROB	Low ROB
**Somalia**								
Nur, 2000 [2]	1995	2.4	42	Convenience	Low precision	Low ROB	High ROB	High ROB
Watts, 1994 [3]	1990	2.3	43	Convenience	Low precision	Low ROB	High ROB	Unclear
Nur, 2000 [2]	1995	7	57	Convenience	Low precision	Low ROB	High ROB	Unclear
Bile, 1993 [4]	1989	40.3	62	Convenience	Low precision	Low ROB	High ROB	Unclear
Bile, 1993 [4]	1989-1989	6.5	62	Convenience	Low precision	Low ROB	High ROB	Unclear
Bile, 1993 [4]	1987	0	76	Convenience	Low precision	Low ROB	High ROB	Low ROB
Watts, 1994 [3]	1990	1.3	79	Convenience	Low precision	Low ROB	High ROB	Unclear
Watts, 1994 [3]	1990	2.5	80	Convenience	Low precision	Low ROB	High ROB	Unclear
Aceti, 1993 [5]	1988-1990	14.6	110	Convenience	Good precision	Low ROB	High ROB	Unclear
Nur, 2000 [2]	1995	0.6	157	Convenience	Good precision	Low ROB	High ROB	Unclear
Aceti, 1993 [5]	1988-1990	2.2	179	Convenience	Good precision	Low ROB	High ROB	Unclear
Watts, 1994 [3]	1990	1.7	236	Convenience	Good precision	Low ROB	High ROB	Unclear
Bile, 1992 [6]	1987	1.1	287	Convenience	Good precision	Low ROB	High ROB	Low ROB
Aceti, 1993 [5]	1988-1990	0	287	Convenience	Good precision	Low ROB	High ROB	Unclear
Bile, 1992 [6]	1987	1.9	309	Convenience	Good precision	Low ROB	High ROB	Low ROB
Aceti, 1993 [5]	1988-1990	1	309	Convenience	Good precision	Low ROB	High ROB	Unclear
EMRO, 2011 [7]	-	0.5	12,759	-	Good precision	Low ROB	Unclear	Unclear
**Sudan**								
Ahmed, 2008 [8]	2007	6.3	16	Convenience	Low precision	Low ROB	High ROB	Unclear
Suliman, 1995 [9]	1994	5.4	37	Convenience	Low precision	Low ROB	High ROB	Unclear
Suliman, 1995 [9]	1994	34.9	46	Convenience	Low precision	Low ROB	High ROB	Unclear
Gadour, 2011[10]	2008	13	62	Simple random	Low precision	Low ROB	Low ROB	Unclear
El-Amine, 2007 [11]	2005	0	62	Convenience	Low precision	Low ROB	High ROB	Low ROB
Nagi, 2007 [12]	2005	1.3	78	Simple random	Low precision	Low ROB	Low ROB	Low ROB

Mohamedani, 2014 [13]	-	4	100	Convenience	Good precision	Low ROB	High ROB	Unclear
Mohamedani, 2014 [13]	-	31.1	106	Convenience	Good precision	Low ROB	High ROB	Unclear
Omer, 2001 [14]	1996-1998	11	115	Convenience	Good precision	Low ROB	High ROB	High ROB
IBBS National Team, 2013 [15]	2011-2012	1	135	Respondent driven	Good precision	Low ROB	Low ROB	Low ROB
IBBS National Team, 2013 [15]	2011-2012	5.9	172	Respondent driven	Good precision	Low ROB	Low ROB	Low ROB
Mudawi, 2007b [16]	2001	4.5	176	Convenience	Good precision	Low ROB	High ROB	Unclear
Omer, 2001 [14]	1996-1998	2	199	Simple random	Good precision	Low ROB	Low ROB	Low ROB
Nail, 2008 [17]	2007	0	211	Convenience	Good precision	Low ROB	High ROB	High ROB
El-Amine, 2007 [11]	2005	23.7	236	Convenience	Good precision	Low ROB	High ROB	Unclear
Elfaki, 2008 [18]	-	0	260	Convenience	Good precision	Low ROB	High ROB	Low ROB
IBBS National Team, 2013 [15]	2011-2012	0	288	Respondent driven	Good precision	Low ROB	Low ROB	Low ROB
IBBS National Team, 2013 [15]	2011-2012	0	291	Respondent driven	Good precision	Low ROB	Low ROB	Low ROB
IBBS National Team, 2013 [15]	2011-2012	0	292	Respondent driven	Good precision	Low ROB	Low ROB	Low ROB
IBBS National Team, 2013 [15]	2011-2012	0	296	Respondent driven	Good precision	Low ROB	Low ROB	Low ROB
IBBS National Team, 2013 [15]	2011-2012	0	296	Respondent driven	Good precision	Low ROB	Low ROB	Low ROB
IBBS National Team, 2013 [15]	2011-2012	5.1	299	Respondent driven	Good precision	Low ROB	Low ROB	Low ROB
IBBS National Team, 2013 [15]	2011-2012	0.6	300	Respondent driven	Good precision	Low ROB	Low ROB	Low ROB
IBBS National Team, 2013 [15]	2011-2012	0.2	303	Respondent driven	Good precision	Low ROB	Low ROB	Low ROB
IBBS National Team, 2013 [15]	2011-2012	2.6	303	Respondent driven	Good precision	Low ROB	Low ROB	Low ROB
IBBS National Team, 2013 [15]	2011-2012	0.5	303	Respondent driven	Good precision	Low ROB	Low ROB	Low ROB
IBBS National Team, 2013 [15]	2011-2012	0.2	304	Respondent driven	Good precision	Low ROB	Low ROB	Low ROB
IBBS National Team, 2013 [15]	2011-2012	0.5	305	Respondent driven	Good precision	Low ROB	Low ROB	Low ROB
IBBS National Team, 2013 [15]	2011-2012	1.5	305	Respondent driven	Good precision	Low ROB	Low ROB	Low ROB
IBBS National Team, 2013 [15]	2011-2012	0.5	305	Respondent driven	Good precision	Low ROB	Low ROB	Low ROB
IBBS National Team, 2013 [15]	2011-2012	1.1	307	Respondent driven	Good precision	Low ROB	Low ROB	Low ROB
IBBS National Team, 2013 [15]	2011-2012	0	312	Respondent driven	Good precision	Low ROB	Low ROB	Low ROB
Gasim, 2012 [19]	2010	8.5	353	Convenience	Good precision	Low ROB	High ROB	Low ROB
Mudawi, 2014 [20]	2010-2012	1.7	358	Convenience	Good precision	Low ROB	High ROB	Low ROB
Osman, 2014 [21]	2011	1.3	396	Convenience	Good precision	Low ROB	High ROB	Unclear

Abou, 2009 [22]	2007	1	400	Convenience	Good precision	Low ROB	High ROB	Low ROB
Mudawi, 2007a [23]	2000	2.2	410	Convenience	Good precision	Low ROB	High ROB	Low ROB
Elsheikh, 2007 [24]	2006	0.6	423	Convenience	Good precision	Low ROB	High ROB	Low ROB
McCarthy, 1994 [25]	1989	3	666	Convenience	Good precision	Low ROB	High ROB	Unclear
**Yemen**								
Haidar, 2002 [26]	1997-1999	40	30	Convenience	Low precision	Low ROB	High ROB	Unclear
Selm, 2010 [27]	2007	62.7	51	Convenience	Low precision	Low ROB	High ROB	Low ROB
Denis, 1994 [28]	1988-1990	3.9	51	Convenience	Low precision	Low ROB	High ROB	Unclear
Al Selwi, 2009 [29]	2004-2007	37	54	Convenience	Low precision	Low ROB	High ROB	Unclear
Selm, 2010 [27]	2007	17.9	67	Convenience	Low precision	Low ROB	High ROB	Unclear
Salem, 2009 [30]	2005-2007	10.7	75	Convenience	Low precision	Low ROB	High ROB	Low ROB
Gunaid, 1997 [31]	-	6.4	78	Convenience	Low precision	Low ROB	High ROB	Unclear
Saeed, 2012 [32]	2008-2010	28.4	88	Convenience	Low precision	Low ROB	High ROB	Low ROB
Sallam, 2003 [33]	1999-2002	5.1	99	Convenience	Good precision	Low ROB	High ROB	Low ROB
Selm, 2010 [27]	2007	0.8	100	Convenience	Good precision	Low ROB	High ROB	Unclear
El Guneid, 1993 [34]	-	21.8	107	Convenience	Good precision	Low ROB	High ROB	Low ROB
Salem, 2009 [30]	2005-2007	17.6	117	Convenience	Good precision	Low ROB	High ROB	Low ROB
Denis, 1994 [28]	1988-1990	21	117	Convenience	Good precision	Low ROB	High ROB	Unclear
Al-Moslih, 2001 [35]	-	4.2	120	Convenience	Good precision	Low ROB	High ROB	Unclear
Al-Shamiri, 2011 [36]	2007-2009	2.1	141	Simple random	Good precision	Low ROB	Low ROB	Unclear
Gray, 1999 [37]	1992	1	158	Convenience	Good precision	Low ROB	High ROB	Unclear
Gray, 1999 [37]	1992	1	175	Convenience	Good precision	Low ROB	High ROB	Unclear
Haidar, 2002 [26]	1997-1999	0.5	200	Convenience	Good precision	Low ROB	High ROB	Unclear
Aman, 2015 [38]	2010-2012	40.2	219	Convenience	Good precision	Low ROB	High ROB	Unclear
El Guneid, 1993 [34]	-	3.3	243	Convenience	Good precision	Low ROB	High ROB	Low ROB
Salem, 2012 [39]	2001-2008	38.2	251	Convenience	Good precision	Low ROB	High ROB	Low ROB
Gray, 1999 [37]	1992	0	253	Convenience	Good precision	Low ROB	High ROB	Unclear
Al-Moslih, 2001 [35]	-	37.1	286	Convenience	Good precision	Low ROB	High ROB	Unclear
El Guneid, 1993 [34]	-	1	294	Convenience	Good precision	Low ROB	High ROB	Low ROB
Scott, 1992 [40]	1988	6	348	Convenience	Good precision	Low ROB	High ROB	Unclear
Al Mansoob, 2013 [41]	2009-2011	14.2	394	Convenience	Good precision	Low ROB	High ROB	Low ROB
Murad, 2013 [42]	2011	8.5	400	Convenience	Good precision	Low ROB	High ROB	Unclear
Bakhubaira, 2009 [43]	2007-2008	8	449	Convenience	Good precision	Low ROB	High ROB	Unclear

Al-Waleedi, 2012 [44]	2007-2008	1.3	469	Simple random	Good precision	Low ROB	High ROB	Low ROB
Sallam, 2003 [33]	1999-2002	0.2	493	Convenience	Good precision	Low ROB	High ROB	Low ROB
Sallam, 2003 [33]	1999-2002	0.6	494	Convenience	Good precision	Low ROB	High ROB	Unclear
Shidrawi, 2004 [45]	-	3.5	546	Convenience	Good precision	Low ROB	High ROB	Unclear
Saghir, 2012 [46]	2009-2010	0.7	564	Convenience	Good precision	Low ROB	High ROB	Low ROB
Al-Jarba, 2003 [47]	-	1.3	576	Convenience	Good precision	Low ROB	High ROB	Unclear
Sallam, 2003 [33]	1999-2002	1.4	593	Convenience	Good precision	Low ROB	High ROB	Low ROB
Haidar, 2002 [26]	1997-1999	8.8	749	Convenience	Good precision	Low ROB	High ROB	Unclear
Saghir, 2012 [46]	2008-2009	0.9	919	Convenience	Good precision	Low ROB	High ROB	Low ROB
Oshaish, 2008 [48]	-	1	1,000	Convenience	Good precision	Low ROB	High ROB	Unclear
Gacche, 2012 [49]	2010-2011	1.3	2,379	Simple random	Good precision	Low ROB	Low ROB	Low ROB
Haidar, 2002 [26]	1997-1999	2	2,434	Convenience	Good precision	Low ROB	High ROB	Unclear
Alodini, 2012 [50]	2010	3	3,000	Convenience	Good precision	Low ROB	High ROB	Low ROB
Omer, 2010 [51]	2007-2008	2	5,826	Convenience	Good precision	Low ROB	High ROB	Unclear
Salem, 2009 [30]	2005-2007	3.5	8,055	Convenience	Good precision	Low ROB	High ROB	High ROB
Salem, 2009 [30]	2005-2007	4.3	20,329	Convenience	Good precision	Low ROB	High ROB	Low ROB

Abbreviations: ROB: risk of bias; EMRO: WHO regional office for the Eastern Mediterranea

Reference list

1. Dray X, Dray-Spira R, Bronstein JA, Mattera D. [Prevalences of HIV, hepatitis B and hepatitis C in blood donors in the Republic of Djibouti]. Med Trop (Mars). 2005;65(1):39-42. Epub 2005/05/21. PubMed PMID: 15903075.
2. Nur YA, Groen J, Elmi AM, Ott A, Osterhaus AD. Prevalence of serum antibodies against bloodborne and sexually transmitted agents in selected groups in Somalia. Epidemiol Infect. 2000;124(1):137-41. Epub 2000/03/18. PubMed PMID: 10722141; PubMed Central PMCID: PMCPmc2810894.
3. Watts DM, Corwin AL, Omar MA, Hyams KC. Low risk of sexual transmission of hepatitis C virus in Somalia. Transactions of the Royal Society of Tropical Medicine and Hygiene. 1994;88(1):55-6. Epub 1994/01/01. PubMed PMID: 8154002.
4. Bile K, Aden C, Norder H, Magnius L, Lindberg G, Nilsson L. Important role of hepatitis C virus infection as a cause of chronic liver disease in Somalia. Scand J Infect Dis. 1993;25(5):559-64. Epub 1993/01/01. PubMed PMID: 7506842.
5. Aceti A, Taliani G, Bruni R, Sharif OS, Moallin KA, Celestino D, et al. Hepatitis C virus infection in chronic liver disease in Somalia. Am J Trop Med Hyg. 1993;48(4):581-4. Epub 1993/04/01. PubMed PMID: 7683179.
6. Bile K, Mohamud O, Aden C, Isse A, Norder H, Nilsson L, et al. The risk for hepatitis A, B, and C at two institutions for children in Somalia with different socioeconomic conditions. Am J Trop Med Hyg. 1992;47(3):357-64. Epub 1992/09/01. PubMed PMID: 1524149.
7. WHO. EMRO Annual HIV STI reporting form SOMALIA 2011 - Hepatitis C. 2011.
8. Ahmed RE, Karsany MS, Adam I. Brief report: acute viral hepatitis and poor maternal and perinatal outcomes in pregnant Sudanese women. Journal of medical virology. 2008;80(10):1747-8. Epub 2008/08/21. doi: 10.1002/jmv.21284. PubMed PMID: 18712815.
9. Suliman SM, Fessaha S, El Sadig M, El-Hadi MB, Lambert S, Fields H, et al. Prevalence of hepatitis C virus infection in hemodialysis patients in Sudan. Saudi journal of kidney diseases and transplantation : an official publication of the Saudi Center for Organ Transplantation, Saudi Arabia. 1995;6(2):154-6. Epub 1995/04/01. PubMed PMID: 18583856.
10. Gadour MOEH, Mohamed BT. HBV, HCV and HIV among patients with Hemophilia in Khartoum - Sudan. Sudan Journal of Medical Sciences. 2011;6(4):233-7.
11. El-Amin HH, Osman EM, Mekki MO, Abdelraheem MB, Ismail MO, Yousif ME, et al. Hepatitis C virus infection in hemodialysis patients in Sudan: two centers' report. Saudi journal of kidney diseases and transplantation : an official publication of the Saudi Center for Organ Transplantation, Saudi Arabia. 2007;18(1):101-6. Epub 2007/01/24. PubMed PMID: 17237901.
12. Nagi AM, Al Tayeb HA, Ahmed MA. Seroprevalence of hepatitis Band C viral infections among blood donors in Shendi, River Nile State, Sudan. Research Journal of Medicine and Medical Sciences. 2007;2(2):122-6.
13. Mohamedani A. Prevalence of Hepatitis C Virus Antibodies in Patients with Schistosomiasis in Gezira State in Central Sudan. Endemic and Emerging Viral Diseases of Priority in the Middle East and North Africa (MENA) - A Scientific Workshop to Promote Research Collaboration; May 2014; Doha, Qatar2014. p. 86.
14. Omer RE, Van't Veer P, Kadaru AM, Kampman E, el Khidir IM, Fedail SS, et al. The role of hepatitis B and hepatitis C viral infections in the incidence of hepatocellular carcinoma in Sudan. Transactions of the Royal Society of Tropical Medicine and Hygiene. 2001;95(5):487-91. Epub 2001/11/15. PubMed PMID: 11706655.
15. IBBS National Team. Integrated bio-behavioral HIV surveillance (IBBS) among female sex workers and men who have sex with men in 15 states of Sudan, 2011-2012. 2013.
16. Mudawi HM, Smith HM, Rahoud SA, Fletcher IA, Babikir AM, Saeed OK, et al. Epidemiology of HCV infection in Gezira state of central Sudan. Journal of medical virology. 2007;79(4):383-5. Epub 2007/02/22. doi: 10.1002/jmv.20780. PubMed PMID: 17311341.
17. Nail A, Eltiganni S, Imam A. Seroprevalence of Hepatitis B and C among health care workers in Omdurman, Sudan. Sudan Journal of Medical Sciences. 2008;3(3):201-5.
18. Elfaki AMH, Eldour AAA, Elsheikh NMH. Sero-prevalence of immunodeficiency virus, hepatitis B and C and syphilis among blood donors at ElObeid Teaching Hospital, West Sudan. Sudan Journal of Medical Sciences. 2008;3(4):333-7.

19. Gasim GI, Hamdan HZ, Hamdan SZ, Adam I. Epidemiology of hepatitis B and hepatitis C virus infections among hemodialysis patients in Khartoum, Sudan. Journal of medical virology. 2012;84(1):52-5. Epub 2011/11/05. doi: 10.1002/jmv.22256. PubMed PMID: 22052648.
20. Mudawi H, Hussein W, Mukhtar M, Yousif M, Nemeri O, Glebe D, et al. Overt and occult hepatitis B virus infection in adult Sudanese HIV patients. International journal of infectious diseases : IJID : official publication of the International Society for Infectious Diseases. 2014;29:65-70. Epub 2014/12/03. doi: 10.1016/j.ijid.2014.07.004. PubMed PMID: 25449238.
21. Osman AMM, Minrghani OA, Gasim GI, Adam I. Hepatitis B Virus, Hepatitis C Virus and Human Immunodeficiency Virus Infection among Pregnant Women in Central Sudan. Sudanese Journal of Medical Sciences. 2014;9(2):91-5.
22. Abou MA, Eltahir YM. Seropositivity of hepatitis B virus and hepatitis C virus dual infection among blood donors in Nyala teaching hospital. Virol J. 2009;6:227. Epub 2009/12/24. doi: 10.1186/1743-422x-6-227. PubMed PMID: 20028507; PubMed Central PMCID: PMCPmc2804613.
23. Mudawi HM, Smith HM, Fletcher IA, Fedail SS. Prevalence and common genotypes of HCV infection in Sudanese patients with hepatosplenic schistosomiasis. Journal of medical virology. 2007;79(9):1322-4. Epub 2007/07/04. doi: 10.1002/jmv.20865. PubMed PMID: 17607776.
24. Elsheikh RM, Daak AA, Elsheikh MA, Karsany MS, Adam I. Hepatitis B virus and hepatitis C virus in pregnant Sudanese women. Virol J. 2007;4:104. Epub 2007/10/26. doi: 10.1186/1743-422x-4-104. PubMed PMID: 17958904; PubMed Central PMCID: PMCPmc2116999.
25. McCarthy MC, el-Tigani A, Khalid IO, Hyams KC. Hepatitis B and C in Juba, southern Sudan: results of a serosurvey. Transactions of the Royal Society of Tropical Medicine and Hygiene. 1994;88(5):534-6. Epub 1994/09/01. PubMed PMID: 7992329.
26. Haidar NA. Prevalence of hepatitis B and hepatitis C in blood donors and high risk groups in Hajjah, Yemen Republic. Saudi Med J. 2002;23(9):1090-4. Epub 2002/10/09. PubMed PMID: 12370719.
27. Selm SB. Prevalence of hepatitis C virus infection among hemodialysis patients in a single center in Yemen. Saudi journal of kidney diseases and transplantation : an official publication of the Saudi Center for Organ Transplantation, Saudi Arabia. 2010;21(6):1165-8. Epub 2010/11/10. PubMed PMID: 21060200.
28. Denis F, Aussel L, Ranger S, Martin P, Itoua-N'Gaporo A, Frommel D, et al. Prevalence of antibodies to hepatitis C virus among patients with leprosy in several African countries and the Yemen. Journal of medical virology. 1994;43(1):1-4. Epub 1994/05/01. PubMed PMID: 7521898.
29. Al-Selwi AHA, Elezzy Y, Al Ghazali J, Hadi S. Association of hepatocellular carcinoma with hepatic viral markers B and C among Yemenis patients at Althawra Hospital Sana'a. Sudan Medical Journal. 2009;4(3):237-42.
30. Salem AK. Prevalence of HCV among Yemeni patients with non-Hodgkin's lymphoma at Al-Thawra teaching hospital. Gulf J Oncolog. 2009;(5):22-9. Epub 2010/01/21. PubMed PMID: 20084782.
31. Gunaid AA, Nasher TM, el-Guneid AM, Hill M, Dayton R, Pal A, et al. Acute sporadic hepatitis in the Republic of Yemen. Journal of medical virology. 1997;51(1):64-6. Epub 1997/01/01. PubMed PMID: 8986951.
32. Saeed NM, Bawazir AA, Al-Zuraiqi M, Al-Negri F, Yunus F. Why is hepatocellular carcinoma less attributable to viral hepatitis in Yemen? Asian Pacific journal of cancer prevention : APJCP. 2012;13(8):3663-7. Epub 2012/10/27. PubMed PMID: 23098451.
33. Sallam TA, Tong CY, Cuevas LE, Raja'a YA, Othman AM, Al-Kharsa KR. Prevalence of blood-borne viral hepatitis in different communities in Yemen. Epidemiol Infect. 2003;131(1):771-5. Epub 2003/09/02. PubMed PMID: 12948378; PubMed Central PMCID: PMCPmc2870019.
34. El Guneid AM, Gunaid AA, O'Neill AM, Zureikat NI, Coleman JC, Murray-Lyon IM. Prevalence of hepatitis B, C, and D virus markers in Yemeni patients with chronic liver disease. Journal of medical virology. 1993;40(4):330-3. Epub 1993/08/01. PubMed PMID: 8228926.
35. Al-Moslih MI, Al-Huraibi MA. Prevalence of hepatitis C virus among patients with liver disease in the Republic of Yemen. East Mediterr Health J. 2001;7(4-5):771-8. Epub 2004/08/31. PubMed PMID: 15332778.
36. Al-Shamiri AH, Al-Taj MA, Ahmed AS. Prevalence and co-infections of schistosomiasis/hepatitis B and C viruses among school children in an endemic areas in Taiz, Yemen. Asian Pac J Trop Med. 2011;4(5):404-8. Epub 2011/07/21. doi: 10.1016/s1995-7645(11)60113-2. PubMed PMID: 21771686.

37. Gray GC, Kassira EN, Rodier GR, Myers MC, Calamaio CA, Gregory M, et al. Remote village survey for agents causing hepatosplenic disease in the Republic of Yemen. Tropical doctor. 1999;29(4):212-9. Epub 1999/12/01. PubMed PMID: 10578634.
38. Aman K, Al-Dubai SA, Aman R, Hawash A, Alshagga M, Kassim S. Prevalence and associated factors of hepatitis C virus infection among renal disease patients on maintenance hemodialysis in three health centers in Aden, Yemen: a cross sectional study. Saudi journal of kidney diseases and transplantation : an official publication of the Saudi Center for Organ Transplantation, Saudi Arabia. 2015;26(2):380-5. Epub 2015/03/12. PubMed PMID: 25758898.
39. Salem AK, Abdulrab A, Alfakeh Y, Aown A. Hepatocellular carcinoma in Yemeni patients: a single centre experience over an 8-year period. East Mediterr Health J. 2012;18(7):693-9. Epub 2012/08/16. PubMed PMID: 22891515.
40. Scott DA, Constantine NT, Callahan J, Burans JP, Olson JG, al-Fadeel M, et al. The epidemiology of hepatitis C virus antibody in Yemen. Am J Trop Med Hyg. 1992;46(1):63-8. Epub 1992/01/01. PubMed PMID: 1311155.
41. Al-Mansoob AS, Salem AK, Selwi.A.H.A. A-, Assamawi A. Risk factors of hepatitis B and C viruses among patients admitted in surgical departments at Al-Thawra Hospital, Sana'a, Yemen. Sudan Medical Journal. 2013;49(3):168-75. Epub December 2013.
42. Murad EA, Babiker SM, Gasim GI, Rayis DA, Adam I. Epidemiology of hepatitis B and hepatitis C virus infections in pregnant women in Sana'a, Yemen. BMC Pregnancy Childbirth. 2013;13:127. Epub 2013/06/14. doi: 10.1186/1471-2393-13-127. PubMed PMID: 23758990; PubMed Central PMCID: PMCPmc3684507.
43. Bakhubaira S. Hepatitis B, C and human immune deficiency virus among cancer patients attending the National Cancer of Public Health Laboratories - Aden. Abstracts of Researches. 2009;13(2).
44. Al-Waleedi AA, Khader YS. Prevalence of hepatitis B and C infections and associated factors among blood donors in Aden City, Yemen. East Mediterr Health J. 2012;18(6):624-9. Epub 2012/08/15. PubMed PMID: 22888620.
45. Shidrawi R, Ali Al-Huraibi M, Ahmad Al-Haimi M, Dayton R, Murray-Lyon IM. Seroprevalence of markers of viral hepatitis in Yemeni healthcare workers. Journal of medical virology. 2004;73(4):562-5. Epub 2004/06/29. doi: 10.1002/jmv.20126. PubMed PMID: 15221900.
46. Saghir SAM, Al-Hassan FM, Alsalahi OSA, Alhariry AEAA, Baqir HS. Frequencies of HBV, HCV, HIV, and syphilis markers among blood donors: A hospital-based study in Hodeidah, Yemen. Tropical Journal of Pharmaceutical Research. 2012;11(1):132-6. doi: http://dx.doi.org/http://dx.doi.org/10.4314/tjpr.v11i1.17. PubMed PMID: 2012100199.
47. Al-Jarba AS, Al-Sayyari WM. Prevalence of hepatitis B virus and hepatitis C virus in health workers in 3 major hospitals in Aden, Republic of Yemen. Saudi Med J. 2003;24(9):1031-2. Epub 2003/09/16. PubMed PMID: 12973499.
48. Oshaish HA, El Shazly H, Elabsii AR. Prevalence of HBS Ag virus, anti-hepatitis C virus and anti-HIV among volunteer blood donor in Taiz private hospital, Yemen Republic. Assiut Medical Journal. 2008;32(3):163-70.
49. Gacche RN, Kaid AM. Epidemiology of viral hepatitis B and C infections in ibb city, yemen. Hepatitis monthly. 2012;12(7):460-2. Epub 2012/09/26. doi: 10.5812/hepatmon.6140. PubMed PMID: 23008727; PubMed Central PMCID: PMCPmc3437458.
50. Alodini A. Prevalence of Hepatitis B Virus (HBV) and Hepatitis C Virus (HCV) Infection among Blood Donors at Al-Thawra Hospital Sana'a City - Yemen. Yemeni Journal for Medical Sciences. 2012;6:16-20.
51. Omer HH. Distribution of Hepatitis B virus, and Hepatitis C virus among blood donors in Aden Blood Center. Abstracts of Researches. 2010;14(1):7.
