# Supplementary material for: Hepatitis C Virus Epidemiology in Djibouti, Somalia, Sudan, and Yemen: Systematic Review and Meta-Analysis
Source: PLoS One. 2016 Feb 22;11(2):e0149966. doi: 10.1371/journal.pone.0149966 (PMC4764686; doi:10.1371/journal.pone.0149966)
Supplement: S4 Table — (PDF) [file pone.0149966.s005.pdf]

S4 Table. Summary of study-level assessment of precision and risk of bias in HCV antibody prevalence measures, as extracted from eligible reports.

	Number of studies (%)
**Precision of estimates**	
Good precision	79 (78.2)
Low precision	22 (21.8)
Missing	0 (0.0)
**Risk of bias by assessment domains**	
HCV ascertainment	
Low risk of bias	101 (100.0)
High risk of bias	0 (0.0)
Unclear	0 (0.0)
Sampling methodology	
Low risk of bias	23 (22.8)
High risk of bias	77 (76.2)
Unclear	1 (1.0)
Response rate	
Low risk of bias	49 (48.5)
High risk of bias	4 (4.0)
Unclear	48 (47.5)
**Overall risk of bias by assessment domains**	
Low risk of bias	
In at least one assessment domain	101 (100.0)
In at least two assessment domains	51 (50.5)
In all three assessment domains	21 (20.8)
High risk of bias	
In at least one assessment domain	75 (74.3)
In at least two assessment domains	8 (7.9)
In all three assessment domains	0 (0.0)
Total number of studies	101
